# On Data-Driven Sparse Sensing and Linear Estimation of Fluid Flows

**DOI:** 10.3390/s20133752

**Published:** 2020-07-04

**Authors:** Balaji Jayaraman, S M Abdullah Al Mamun

**Affiliations:** School of Mechanical and Aerospace Engineering, Oklahoma State University, Stillwater, OK 74078, USA; smamun@ostatemail.okstate.edu

**Keywords:** sparse reconstruction, extreme learning machines, sensors, SVD, POD, compressive sensing

## Abstract

The reconstruction of fine-scale information from sparse data measured at irregular locations is often needed in many diverse applications, including numerous instances of practical fluid dynamics observed in natural environments. This need is driven by tasks such as data assimilation or the recovery of fine-scale knowledge including models from limited data. Sparse reconstruction is inherently badly represented when formulated as a linear estimation problem. Therefore, the most successful linear estimation approaches are better represented by recovering the full state on an encoded low-dimensional basis that effectively spans the data. Commonly used low-dimensional spaces include those characterized by orthogonal Fourier and data-driven proper orthogonal decomposition (POD) modes. This article deals with the use of linear estimation methods when one encounters a non-orthogonal basis. As a representative thought example, we focus on linear estimation using a basis from shallow extreme learning machine (ELM) autoencoder networks that are easy to learn but non-orthogonal and which certainly do not parsimoniously represent the data, thus requiring numerous sensors for effective reconstruction. In this paper, we present an efficient and robust framework for sparse data-driven sensor placement and the consequent recovery of the higher-resolution field of basis vectors. The performance improvements are illustrated through examples of fluid flows with varying complexity and benchmarked against well-known POD-based sparse recovery methods.

## 1. Introduction

The challenge of multiscale flow sensing lies in the use of fewer sensors than there are scales. Therefore, deciphering the true multiscale behavior of the system is often accomplished through post-processing. In the case of simulations, the sensor (grid points) budgets are limited by computational considerations; therefore, it is necessary to resort to coarse-grained models which in turn are expected to produce nearly accurate outcomes as full-resolution models. Such a situation is commonly encountered in atmospheric turbulence sensing closer to the surface—in a region called the atmospheric boundary layer—where simulation-based research [1,2] has served as a key enabler for the extraction of the explainable knowledge of coherent structures and underlying mechanisms. In recent times, a major driver for the direct measurement of atmospheric turbulence data has been the use of swarms of unmanned vehicles [3,4] flying in the atmosphere, whose capability to extract the wind velocity vectors [5,6] and turbulent statistics of the atmospheric boundary layer [7] have been demonstrated using simulations. This current numerical exploration from our group is one step towards the ultimate goal of the sparse sensing of turbulent fields using unstructured measurements within large flow fields leveraging unmanned aerial vehicle dynamics. In such practical situations as those discussed above, measurement data represent the absolute truth and are often acquired from very few probes, limiting their in-depth analysis. A common recourse is to combine such sparse measurements with physics-based priors, either in the form of idealized simulations (data assimilation), phenomenology or knowledge of a sparse basis to recover detailed information (sparse recovery).

A second example arises from situations (e.g., computational simulations) in which the data are often in surplus and consequently offer the best avenue for the in-depth analysis of realistic flows due to the high density of computational grid probes. With the growth in computing power and the resulting ability to generate big data, it is easy to recognize the need for rapid low-dimensional analysis tools [8,9,10,11,12] and evolutionary models [12,13,14,15,16] and regenerate the high-dimensional state without a significant loss of information [17]. Thus, tools for encoding information into a low-dimensional feature space complement sparse recovery tools that decode compressed information. This, in essence, is a key aspect of leveraging machine learning for fluid flow analysis [18,19] and broadly speaking fo the recovery of coarse-grained information [20].

This work focuses on the algorithmic aspects of recovering a high-dimensional field from sparse data through data-informed sensor placement for accurate reconstruction of the full system state in situations such as those listed above. Although their deployment in practical settings is not demonstrated here, the underlying principles are expected to guide users of the technology.

Regarding related work on linear estimation in the basis space, at a conceptual level, sparse recovery is deeply connected to compressive sensing (CS) [21,22,23,24] which has made it possible to directly sample [18] data in real-time without having to collect high-resolution information and then perform downsampling. Of course, in the case of direct sampling, the recovery algorithm needs a generic or data-driven basis in which the data are sparse. The recovery of fine-scale information from sparse data has been gaining traction in various manifestations including gappy proper orthogonal decomposition (GPOD) [25,26], Fourier-based compressive sensing (CS) [21,22,23,24] and Gaussian kernel-based kriging [27,28,29]. A tangential application of such ideas is in the acceleration of nonlinear model order reduction using sparse sampling for hyper-reduction [30,31,32,33]. Sparse recovery techniques such as GPOD [25,26,30] utilize the knowledge of the POD basis computed offline from the data ensemble to recast the reconstruction problem in the feature space and solve it using least-squares minimization approaches. Derivatives [25,27,30,34] of this approach include an iterative formulation [25,27,30,34] to successively approximate the POD basis in the event that the low-dimensional basis is not known a priori. Nevertheless, these iterative approaches remain impractical on account of their limited accuracy and computational cost.

While data-driven POD-based approaches can optimally represent the data, they do not generalize well. Therefore, their use in practice requires a priori knowledge of the basis vectors. One way to overcome this stringent requirement is to adopt computational simulations of the twin dynamical system or model simulations to build the basis library. Nevertheless, such methods find tremendous value in data-driven modeling (machine learning, Koopman operator models [13,35]) applications and the nonlinear model order reduction [10] of systems that are statistically stationary.

Alternatively, one can use a generic basis such as Fourier or wavelets that may not always be effective at dimensionality reduction on a data-driven basis, especially for inhomogeneous fluid flow phenomena with multiple scales and sharp gradients. The resulting higher-dimensional feature space requires more sensors for accurate reconstruction. Consequently, such flow systems are invariably under-sampled during sensing, partially due to the algorithm. To recover the higher-dimensional state, the best sparse solution is often sought instead of a least-squares estimate that overfits to the undersampled data. The success of compressive sensing (CS) [21,22,23,24] lies in achieving this using $l_{1}$-norm regularized least-squares reconstruction.

Sparsity-promoting $l_{1}$ regularized reconstruction can also be combined with a data-driven POD basis, such as in the reconstruction of sparse flow fields from particle image velocimetry (PIV) data [18] and pressure measurements around a cylinder surface [19]. Thus the choice of basis has an impact on algorithmic design.

Regarding sensor placement and sparse recovery, in addition to the choice of the basis space and its relationship with the inversion algorithm, the choice of sparse measurement locations impacts sparse recovery. The sparse measurement locations determine what information pertaining to the physical system is collected and in turn determines the quality of the sparse recovery. In general, identifying “optimal” sensing locations for spatio-temporal fields is an NP-hard problem and an open topic of research. However, greedy smart sampling methods have been reported in the literature such as using extrema of POD-basis vectors [36,37], hyperreduction approaches such as DEIM for sensing [31,38] and objective-based matrix condition number minimization (or maximization, as the case may be) using both explicit [26,39] and submatrix volume maximization using QR-pivoting [40]. All these methods have primarily been employed with simulation or experimental (using particle image velocimetry (PIV)) data, where the distributed information of the field is available to identify sensor placement. In addition, there is a vast amount of interesting literature on the greedy sensing of network dynamics with discrete events where extreme event detection, such as faults, is required; for example, water [41,42,43] or communication networks. Given that the interest in this paper is “super-resolution” or the sparse recovery of continuous fields from sparse measurements, we focus on techniques such as DEIM and QR-pivoting-based matrix conditioning.

### Contribution of This Work

In this article, we explore the use of an arbitrary data-driven basis for sensor placement and sparse recovery applications. Such situations may be encountered in machine learning applications where basis spaces that do not optimally span the data may be readily available from other stages of the data science workflow. An example of such a basis is the modes from dynamic mode decomposition (DMD) [35] or projections available in extreme learning machine (ELM)-based autoencoders [44,45,46], among others. Such DMD and ELM modes are known to be non-orthogonal, unlike POD-modes, and their suitability for data-driven sensor placement/sparse recovery has not been explored to our knowledge. Further, the arbitrary non-orthogonal basis suffers from a lack of parsimony for low-dimensional representation and a lack of inherent hierarchy, resulting in larger sensor budgets, inaccurate reconstruction, ineffective sensor placement due to basis non-orthogonality and the enhanced complexity of the inverse problem solution. To this end, we develop a framework that combines the Gram–Schmidt orthogonalization of the arbitrary data-driven basis with well known methods for data-driven sensor placement and linear sparse estimation.

We systematically analyze the accuracy of this integrated sparse reconstruction (SR) framework by comparing it with the corresponding POD-based SR—a standard approach for the linear sparse estimation of fluid flows. The analysis focuses on comparing the basis structure, the basis dimension for a chosen representation accuracy, the basis hierarchy for the chosen datasets and the interplay of SR accuracy with sensor budget and placement. In particular, the effect of sensor placement on sparse recovery has barely been explored in the literature and provides an insight into the practical limitations of sparse recovery design. In this way, the current effort builds on our earlier research [47,48] that characterized this interplay. For this study, we chose two use cases to demonstrate the methods: a low-dimensional cylinder wake flow at a laminar Reynolds number (Re=100) and higher dimensional sea surface temperature field from NOAA.

The rest of manuscript is organized as follows. In Section 2, we review the basics of sparse reconstruction theory and different choices of data-driven bases including POD and ELM. Section 3 discusses the role of measurement locations and reviews the approaches for data-driven sensor placement. In Section 4 and Section 6, we summarize the different algorithms employed for SR and training data generation. Section 7 compares the structure of the different data-driven bases, while Section 8 compares their performance for the different use cases. We summarize the major conclusions from this study in Section 9.

## 2. Recovering Resolved Fields from Sparse Data Using Linear Estimation

For certain high-resolution data of fluid flow at any particular instant, $x\in R^{N}$, the corresponding sparse representation may be written as $\tilde{x}\in R^{P}$ with P≪N. Then, the sparse reconstruction problem is to recover *x* given $\tilde{x}$ along with information of the sensor locations in the form of the measurement matrix $C\in R^{P\times N}$ as

(1) $$\tilde{x}=Cx.$$

Often, in practice, it is only the sensor locations that are available; therefore, an imaginary reconstruction grid may be designed to suit the desired end goals. In this way, the measurement matrix C shows how the sparse data $\tilde{x}$ (of dimension *P*) are downsampled from better-resolved sensor data, *x* (of dimension *N*). In this article, we focus on vectors *x* that have a sparse representation in a basis space $\Phi \in R^{N\times K}$ such that K≪N and yielding x=Φa. Naturally, the recovery of lost information is never absolute, as the reconstruction problem is ill-posed; i.e., there are more unknowns than equations in Equation (1), which rules out least-squares solutions such as $x=C^{+}\tilde{x}$.

### 2.1. Sparse Reconstruction Theory

Sparse reconstruction has theoretical foundations in inverse problem frameworks [49] applied to diverse fields such as geophysics [50,51] and image processing [52]. Many signals tend to be “compressible” or sparse in some *K*-sparse bases Φ; i.e.,

(2) $$x=\sum_{i=1}^{N_{b}}\phi_{i}a_{i}\;\;\;\;\mathrm{or}\;\;\;\;x=\Phi a,$$

where $\Phi \in R^{N\times N_{b}}$ and $a\in R^{N_{b}}$ with *K* significant or non-zero elements. In general, *K* is not known a priori for an unknown system with only sparse data available. Further, it is not always obvious which *K* of the $N_{b}$ basis vectors $\phi_{i}$ results in the most accurate reconstruction. A prudent and common approach is to adopt a more exhaustive basis set of dimension $N_{b}\approx P>K$ for a desired *K*, all of which will be naturally smaller than the dimension *N* of the full-field data, and then to search for the optimal *K*-sparse solution. In practice, it makes sense to have $K,N_{b}\ll N$, especially if the choice of basis is optimal, such as for data-driven POD modes. Therefore, the choice of $\Phi ,K,N\mathrm{and}N_{b}$ represents the overall problem design. While standard image compression techniques (with transform coding in JPEG and JPEG-2000 compression standards [53]) adopt a sample-and-then-compress approach—i.e., they collect a high-resolution sample, transform it to a Fourier or wavelet basis space and retain only a suitable *K*-sparse structure—techniques such as compressive sensing [21,23,54,55,56] and sparse reconstruction [12,17,26,39,48] directly infer the *K*-sparse coefficients by essentially combining the steps in Equations (1) and (2) as below:

(3) $$\tilde{x}=C\Phi a=\Theta a,$$

where $\Theta \in R^{P\times N_{b}}$ relates the basis coefficients *a* in the feature space and the sparse data $\tilde{x}$ in the physical space. The challenge of recovering x from the underdetermined system in Equation (1) arises from C being ill-conditioned and N≫P. However, when *x* is sparse in Φ, the recovery of $a\in R^{K}$ using Equation (3) becomes feasible as K∼P; that is, solving for *K* unknowns (in *a*) using *P* constraints ($\tilde{x}$), as per Equation (6). Commonly, the *s*-norm regularized least squares error *s* is minimized, which is chosen appropriately to recover *x* as per Equation (2). The $l_{2}$-regularized method estimates *a* such that the expression in Equation (4) is minimized.

(4) $$∥\tilde{x}{-\Theta a∥}_{2}^{2}+\lambda {∥a∥}_{2}^{2}.$$

The exact expression for *a* uses the left pesudo-inverse of Θ, as given in Equation (4),

(5) $$a={\left(\Theta \right)}^{+}\tilde{x},$$

where $\Theta^{+}={\left(\Theta^{T}\Theta +\lambda I\right)}^{-1}\Theta^{T}\tilde{x}$. This regularized least-squares approach is nearly identical to the GPOD algorithm of Everson and Sirovich [30] when Φ is the POD basis. However, $\tilde{X}$ in GPOD contains zeros as placeholders for all the missing elements, whereas the above formulation retains only the measured data points. A possible method to enhance the sparsity of the resulting *a* is to minimize $∥a^{\prime }{∥}_{0}$; i.e., minimize the number of non-zero elements such that $\Theta a^{\prime }=\tilde{x}$. It has been shown [57] that P=K+1 (P>K in general) independent measurements are sufficient to recover the sparse coefficients with high probability using $l_{0}$ reconstruction. On the other hand, when employing P≤K independent measurements, the probability of recovering the sparse solution is diminished. Compressed sensing [58,59,60,61] overcomes the computational difficulties with NP-complex $l_{0}$-reconstruction using $l_{1}$ methods that guarantee the near-exact recovery of *K*-sparse coefficients. The reconstruction of $l_{1}$ is a relatively simple convex optimization problem as compared to $l_{0}$ and solvable using linear programming techniques such as basis pursuit [21,54,62], shrinkage [63] and sequential thresholded least-squares approaches [64]. These different methods solve the constrained reconstruction problem in Equation (6) with complexity $O(N^{3})$ for $N_{b}\approx N$ at the cost of needing $P>O(Klog\left(N_{b}/K\right))$ measurements [21,54,58] to exactly reconstruct the *K*-sparse vectors.

(6) $$\begin{array}{cc} \; & l_{1}\;\;\mathrm{reconstruction}:\;a=\min\;\;\;∥a^{\prime }{∥}_{1}\;\mathrm{such}\;\mathrm{that}\;\Theta a^{\prime }=\tilde{x}\\ \; & l_{1}\;\mathrm{cost}\;\mathrm{function}\;\mathrm{to}\;\mathrm{minimize}:\min∥\tilde{x}{-\Theta a∥}_{2}^{2}+\lambda {∥a∥}_{1} \end{array}$$

As reported in prior efforts [47,48], the interplay between the design choices of $N_{b},K,P$ and the choice of algorithm are non-trivial and impact the reconstruction quality. For clarity, $N_{b}$ is the candidate basis dimension, meaning that $N_{b}⪅N$ and *K* is the desired system reconstruction dimension, which determines the best possible sparse recovery quality; *P* is the available sensor budget. Often, P≥K is required for reasonably accurate sparse recovery. In addition to the sensor budget, the sensor placement also plays an important role as it is tied to the structure of individual basis functions in Φ to determine the condition of Θ. Therefore, it makes sense to ensure the sensor–basis vector relationship helps improve the sparse recovery quality. Often, this involves placing sensors in such a way that the measurement basis (rows of *C*) is incoherent with the data basis Φ. Smart sensor placement strategies provide a more structured approach by taking into account the underlying physics and coherence of the data.

### 2.2. Computation of Data-Driven Basis

Given the central role that basis choice plays in the sparse recovery of continuous fields, especially with limited data, it is important to consider $\phi_{i}$s that are customized to the data. Among other factors, such as the carrying signatures of the physical phenomena, this also results in a parsimonious representation of the data. In fact, it has been shown [18] that the data-driven POD basis outperforms the generic cosine basis when performing reconstruction with small amounts of data, while the accuracy becomes comparable with more data. In this work, we explore two classes of data-driven spaces, namely POD and extreme learning machine (ELM) bases, with POD-based SR serving as a benchmark for data-driven SR.

#### Proper Orthogonal Decomposition (POD) Basis

Proper orthogonal decomposition (POD) is a popular approach for dimensionality reduction. The POD modes are computed from the eigendecomposition of the symmetric, positive, definite two-point spatial (or temporal) correlation tensor of the data snapshots. The appropriately scaled eigenvectors represent the singular vectors or POD modes in space or time—as the case may be—for a given dataset. These POD modes or singular vectors form an orthogonal basis that optimally represents the data snapshots in terms of the least-squares approach. Therefore, the detailed flow field can be reconstructed using only a few (relative to the system state dimension) coefficients; therefore, this is attractive for dimensionality reduction. Obviously, not all systems have a fast decaying singular value spectrum; therefore, the extent of dimension reduction is problem-dependent. Given that fluid mechanics problems typically have a much larger state dimension than the number of snapshots (N≫M), the POD problem is reformulated as the eigendecomposition of the two-point temporal correlation tensor of dimension M×M [65]. Denoting the full state data snapshots as $X\in R^{N\times M}$ (different from $x\in R^{N}$) where N,M are the state and snapshot dimensions, the symmetric temporal correlation matrix ${\bar{C}}_{M}\in R^{M\times M}$ (Equation (7)) can be built and the eigendecomposition performed as shown in Equation (8).

(7) $${\bar{C}}_{M}=X^{T}X.$$

(8) $${\bar{C}}_{M}V=V\Lambda .$$

where $V=[v_{1},v_{2},\cdots ,v_{M}]$ represent the eigenvectors and the diagonal elements of Λ denote the eigenvalues $\left[\lambda_{1},\lambda_{2},\cdots ,\lambda_{M}\right]$. The POD/singular value decomposition (SVD) modes Φ and coefficients a (for the linear expansion as shown in Equation (2)) can then be estimated as per Equations (9) and (10).

(9) $$\Phi =XV\sqrt{\Lambda^{-1}}.$$

(10) $$a=\Phi^{T}X.$$

The method of snapshots limits the maximum number of POD basis vectors to *M*, which is typically smaller than the dimension of full state vectors, *N*. Further dimension reduction may be achieved using singular value thresholding such that K<M modes are retained.

### 2.3. ELM Autoencoder Basis

**Figure 1:**
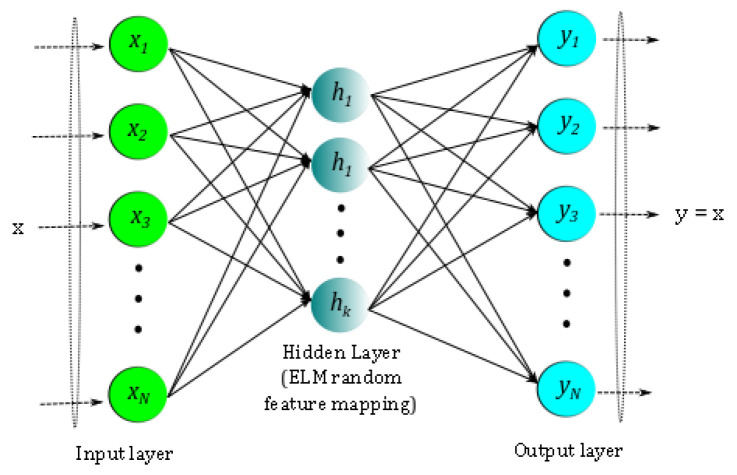
Schematic of the extreme learning machine (ELM) autoencoder network. In this architecture, the output features are the same as input features.

In this paper, we explore methods for dealing with unconventional data-driven bases that are commonly encountered in sparse data-driven modeling. For example, it is not uncommon to adopt radial basis functions (RBFs) to generate continuous representations of discrete measurements due to their suitability for representing a wide variety of unknown flow physics [11]. In this work, we leverage bases generated from extreme learning machines (ELMs) [44,45]—a class of shallow neural network regressors employing a Gaussian prior that was used as encoder–decoder maps for a given data set by Zhou et al. [46,66,67]. The ELM-autoencoder is a single hidden-layer feedforward neural network (SLFN) with randomized projection followed by the Gaussian activation of the data onto hidden nodes and a linear map to the output (the same as the input). By setting the number of hidden nodes to a small fraction of the input/output feature dimension, we generate sparse representations of the state, as shown in Figure 1. Given snapshots of data $X\in R^{N\times M}$ (or simply $x_{j}\in R^{N}$ for j=1⋯M), we relate the full state data to a *K*-dimensional feature space vector using the ELM autoencoder, as shown below in Figure 1 and Equation (11).

(11) $$x_{j}=\sum_{i=1}^{K}\phi_{i}a_{i}^{j}=\sum_{i=1}^{K}\phi_{i}h_{i}\left(x_{j}\right)=\sum_{i=1}^{K}\phi_{i}g\left(w_{i}^{T}x_{j}+b_{i}\right)$$

where $x_{j}\in R^{N}$ is a snapshot of the input data with *j* as the snapshot index, $w_{i}\in R^{N}$ is the random input weight vector, $b_{i}$ is the random bias, g(.) is the activation function (chosen as the Gaussian; i.e., $g\left(z\right)=e^{-(z^{2})}$) operating on the linearly transformed input state to yield and $h_{i}$ and $\phi_{i}\in R^{N}$ ($\Phi \in R^{N\times K}$) are the weights that map hidden layer features to the output. In matrix form, the linear Equation (11) can be written as in Equation (12), where a is the matrix of outputs (with elements $a_{i}^{j}$) from the hidden layer and $h\left(x_{j}\right)=\left[h_{1}\left(x_{j}\right),\cdots ,h_{k}\left(x_{j}\right)\right]=\left[a_{1}^{j},\cdots ,a_{k}^{j}\right]$, which represents the output corresponding to the input snapshot $x_{j}$.

(12) $$X=\Phi a;a={\left[\begin{array}{ccc} a_{1}^{1} & \cdots & a_{k}^{1}\\ \vdots & \ddots & \vdots \\ a_{1}^{M} & \cdots & a_{k}^{M} \end{array}\right]}^{T}={\left[\begin{array}{c} h(x_{1})\\ \vdots \\ h(x_{M}) \end{array}\right]}^{T}={\left[\begin{array}{ccc} h_{1}\left(x_{1}\right) & \cdots & h_{k}\left(x_{1}\right)\\ \vdots & \ddots & \vdots \\ h_{1}\left(x_{M}\right) & \cdots & h_{k}\left(x_{M}\right) \end{array}\right]}^{T}$$

The output weights in matrix form for a given X are shown in Equation (13).

(13) $$X={\left[\begin{array}{ccc} x_{1}^{1} & \cdots & x_{N}^{1}\\ \vdots & \ddots & \vdots \\ x_{1}^{M} & \cdots & x_{N}^{M} \end{array}\right]}^{T};\Phi ={\left[\begin{array}{ccc} \phi_{1}^{1} & \cdots & \phi_{1}^{N}\\ \vdots & \ddots & \vdots \\ \phi_{k}^{1} & \cdots & \phi_{k}^{N} \end{array}\right]}^{T}$$

Using Equation (12), **Φ** is estimated in a least squares sense as in Equation (14).

(14) $$\Phi_{train}=\Phi =Xa^{+}=Xa_{train}^{+}$$

The columns of Φ represent the ELM-basis, and the density of the hidden layer determines the effective system dimension. However, a major drawback of this basis is the lack of orthogonality. It is well known that orthogonal bases yield parsimonious representations of the data as compared to their non-orthogonal counterparts, therefore requiring fewer sensors for a similar reconstruction quality [47]. In addition, basis orthogonality is useful for data-driven sensor placement using methods such as discrete empirical interpolation method (DEIM) [31]. To this end, we extend the ELM basis generation with a Gram–Schmidt procedure (Algorithm 1) to generate an orthogonal $\Phi^{ELM-GS}$ which spans more or less the same subspace as $\Phi^{ELM}$. This particular step represents a one-time cost but can result in greatly improved properties for sparse recovery, as will be seen from the results presented in the later sections.

**Algorithm 1:** Gram–Schmidt Orthogonalization of ELM Basis (ELM-GS) **input**: N dimensional non-orthogonal basis $\left[\Phi_{1},\Phi_{2},\ldots \Phi_{K}\right]$ **output**: N dimensional orthogonal basis $\left[\Phi_{1}^{orth},\Phi_{2}^{orth},\ldots \Phi_{K}^{orth}\right]$

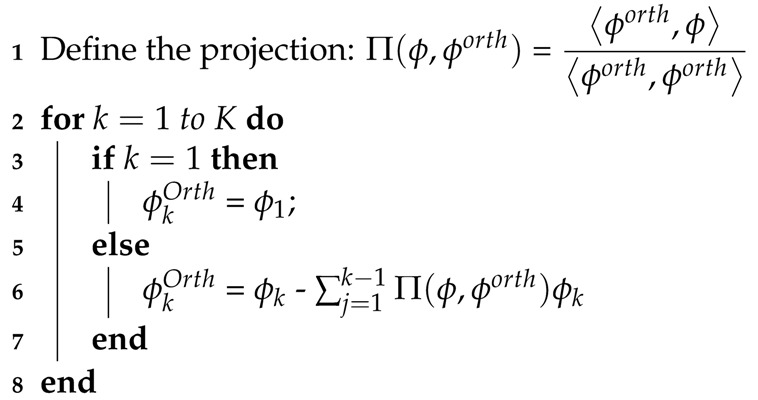



## 3. Sensor Placement, Data Basis and Incoherence

**Figure 2:**
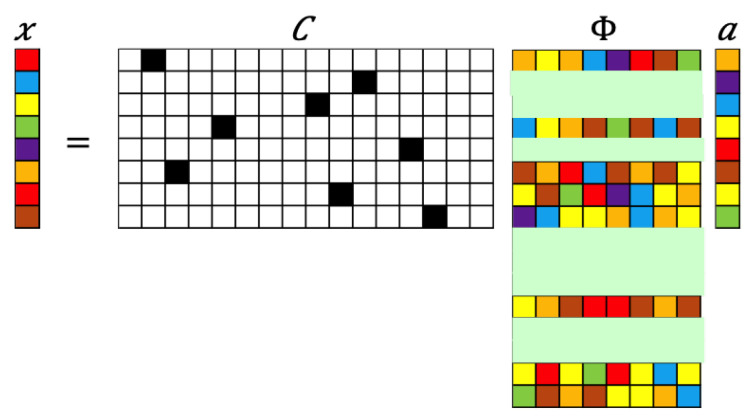
Schematic illustration of sparse sensor placement. The pastel-colored rectangles represent rows activated by the sensors denoted in the measurement matrix through dark squares.

It is well known that recovery quality is tied to sensor placement (structure of measurement matrix, C), budget and the choice of basis, Φ [48]. Specifically, the sensor placement needs to be incoherent with respect to the low-dimensional basis Φ [23], and this is usually accomplished by using a randomized measurement matrix for Φ. In this study, we restrict ourselves to single-pixel measurements with C of the form $C\leftarrow {\left[e_{\varrho_{1}},e_{\varrho_{2}},\cdots ,e_{\varrho_{p}}\right]}^{T}$, where $e_{\varrho_{p}}$ is column vector with zeros and a value of one at the sensor index *p*. The purpose of making C (Equations (1)–(3)) incoherent with respect to the basis Φ is to ensure that the measurements distributed in space excite the different modes and ensure CΦ is not rank-deficient. This is usually quantified in terms of the coherency number, μ, as shown in Equation (15) [68],

(15) $$\mu \left(C,\Phi \right)=\sqrt{N}\cdot \max_{i\leq P,\;j\leq K}\left|\langle c_{i},\Phi_{j}\rangle \right|,$$

where $c_{i}$ is a row vector in C (i.e., $c_{i}=e_{\varrho_{j}}$) and $\phi_{j}$ is a column vector of Φ. μ which typically ranges from 1 (incoherent) to $\sqrt{N}$ (coherent). The smaller the μ, the fewer measurements are needed to reconstruct the data. There are many metrics that can be leveraged for improving sensor placement. However, identifying a truly optimal sensor arrangement is combinatorially hard and therefore an active area of research. There is a current search for greedy sensor placement algorithms with near-optimal performance by leveraging a variety of optimization surrogates [26,37,69,70]. In the context of flow reconstruction, sensor placement can be viewed as a problem of identifying and activating only a few rows of the basis matrix Φ such that the matrix Θ (for $P=K=N_{b}$) or its variants $M=\Theta^{T}\Theta$ or $M=\Theta \Theta^{T}$ (depending on if $P>K=N_{b}$ or $P<K=N_{b}$ respectively) have low condition numbers, as schematically illustrated in Figure 2.

In this study, we consider two different greedy approaches for nearly optimal sensor placement in sparse recovery applications, namely the discrete empirical interpolation method (DEIM) [31,71] and reduced matrix QR-factorization with column pivoting [40] instead of choosing sensors at random locations within the region. These approaches are summarized below for completeness.

The most simple and efficient sensor placement strategy is to sample at random locations by choosing the first *P* values from a random permutation of the entire sensor array of dimension *N*. Several ideas can also be adopted, such as *K*-means clustering, as was used in [12].

Sensors generated from the pivot matrix in QR factorization (with column pivoting) are designed to minimize the condition number of the matrix Θ or $M=\Theta \Theta^{T}$ to improve the full state recovery. Specifically, the reduced matrix QR factorization [72] decomposes any given real matrix $A\in R^{S\times T}$ with a full column rank into a unitary matrix $Q\in R^{S\times T}$ and an upper triangular matrix $R\in R^{T\times T}$. QR factorization with column pivoting yields AD=QR, with $D\in R^{T\times T}$ being a square column permutation matrix containing ones and zeros such that the diagonal values of R, $r_{ii}$ form a decreasing sequence. Therefore, choosing the first *P* columns of A and first *P* rows of D maximizes the determinant of the submatrix AD for a given budget *P*. Given that the measurement matrix C selects columns of $\Phi^{T}$ (or rows of Φ) and interpreting AD as $\Theta^{T}=\Phi^{T}C^{T}$, the connection between the permutation matrix D and the measurement matrix C can be directly observed. Using $C=D^{T}$ ensures that the submatrix volume of Θ is maximized and its condition number minimized. We refer the reader to the work presented in [40,48] for a more detailed discussion of the algorithm.

In contrast, the discrete empirical interpolation method (DEIM) [31,71] iteratively tests the linear dependence of the columns of Θ=CΦ to identify each sensor location. Here, we identify interpolation points (with indices $\varrho_{j}$) with the most linear dependence error relative to previously determined interpolation points. The primary idea behind DEIM is to estimate a high-dimensional state using information at sparsely sampled interpolation points which can be adopted for sensor placement in sparse recovery. While the sequence of input bases is not critical for the QR-pivoting based approach, it is important for DEIM. Therefore, the sensor placement will depend on basis choice. Secondly, the orthogonality of the basis ensures the interpolation indices are hierarchical and non-repeating. Therefore, the sensor placement methods are not as effective with non-orthogonal bases.

## 4. Sparse Recovery Algorithm

In addition to basis generation and data-driven sensor placement, the choice of linear estimation approaches is also critical (Section 2.1). This choice depends on the combination of sensor budget and basis dimension. In this work, we adopt the $l_{2}$ sparse reconstruction (summarized through Equations (3) and (4)) with K≤M basis vectors (Φ), which is also the dimension of the feature vector *a*. Least-squares reconstruction demands the candidate basis dimension, $N_{b}$, be the same as *K*, the reconstruction dimension. The naming convention adopted is as follows: $x_{j}\in R^{N}$ denotes the instantaneous $j^{th}$ full flow state with the entire dataset of *M* snapshots denoted by $X\in R^{N\times M}$. The algorithm used in this work applies to both single and batch-style reconstruction in series and parallel.

One can construct the measurement matrix—i.e., $C\in R^{N\times N}$ or $C\in R^{P\times N}$—depending on the dimension of the sparse data vector; that is, whether ${\tilde{x}}_{j}\in R^{N}$ or $R^{P}$. In this work, we consistently use the high-dimensional version of ${\tilde{x}}_{j}$ which is similar to the earlier work on gappy POD methods [30]. For high-resolution data $x_{j}\in R^{N}$ with a chosen basis of $\phi_{k}\in R^{N}$, the low-dimensional features, $a^{j}\in R^{K}$, are obtained as per Equation (16). We also define the masked (incomplete) data ${\tilde{x}}_{j}\in R^{N}$, corresponding measurement matrix $C\in R^{N\times N}$ and mask vector $m\in R^{N}$. Since the GPOD results in a larger measurement matrix (N×N) with numerous rows of zeros, the mask vector (containing 1s and 0s) bypasses the added computational complexity by operating on $x_{j}$ through a point-wise multiplication operator <·>; i.e., ${\tilde{x}}_{j}=<m_{j}\;\cdot \;x_{j}>$, where each element of $x_{j}$ multiplies with the corresponding element of $m_{j}$. This compact representation allows the $m_{j}$ to be different for each snapshot if desired.

(16) $$x_{j}=\sum_{k=1}^{K}\phi_{k}a_{k}^{j};{\tilde{x}}_{j}=<m\;\cdot \;x_{j}>=Cx_{j}.$$

In SR, we recover the full data from the masked data given in Equation (17) by estimating the coefficients ${\bar{a}}^{j}$ (in the $l_{2}$ sense) with the basis $\phi_{k}$ generated offline. As the masked basis vectors are not necessarily orthogonal, the coefficient vector ${\bar{a}}^{j}$ is approximated by minimizing the least-squares error $E_{j}$ (Equation (18)).

(17) $${\tilde{x}}_{j}\approx m\sum_{k=1}^{K}{\bar{a}}_{k}^{j}\phi_{k}.$$

(18) $$E_{j}={\left|\left|{\tilde{x}}_{j}-m\sum_{k=1}^{K}{\bar{a}}_{k}^{j}\phi_{k}\right|\right|}_{2}^{2}={\left|\left|{\tilde{x}}_{j}-m\;\cdot \;\Phi {\bar{a}}^{j}\right|\right|}_{2}^{2}={\left|\left|{\tilde{x}}_{j}-C\Phi {\bar{a}}^{j}\right|\right|}_{2}^{2}={\left|\left|{\tilde{x}}_{j}-\sum_{k=1}^{K}{\bar{a}}_{k}^{j}{\tilde{\phi }}_{k}\right|\right|}_{2}^{2}.$$

where *m* is multiplied point-wise with each column of Φ to yield ${\tilde{\phi }}_{k}$. The above formulation is valid for a case in which the measurement locations are static. In the case of the dynamically evolving sensor placement, the mask vector $m_{j}$ changes with every snapshot ${\tilde{x}}_{j}$ for j=1..M. The error $E_{j}$ represents the individual snapshot reconstruction error that is be minimized to estimate the features ${\bar{a}}^{j}$. It is easily seen that one has to minimize the different $E_{j}$s separately to estimate the entire coefficient matrix, $\bar{a}\in R^{K\times M}$ for the entire batch of snapshots. In the above formulation, $\tilde{\Phi }$ is analogous to CΦ=Θ in Equation (3).

To minimize $E_{j}$, its derivative is computed with respect to ${\bar{a}}_{j}$ resulting in the normal equation given by $M{\bar{a}}^{j}=f^{j}$ where $M_{k1,k2}=\langle {\tilde{\phi }}_{k1},{\tilde{\phi }}_{k2}\rangle$ or $M={\tilde{\Phi }}^{T}\tilde{\Phi }$ and $f_{k}^{j}=\langle {\tilde{x}}_{j},{\tilde{\phi }}_{k}\rangle$ or $f^{j}={\tilde{\Phi }}^{T}{\tilde{x}}_{j}$. The recovered solution is given by ${x^{SR}}_{j}=\sum_{k=1}^{K}\phi_{k}{\bar{a}}_{k}^{j}$.

### 4.1. Sequential Thresholding for $l_{1}$ Regularized Least Squares

Two situations commonly need to be handled: (i) a case with very few sensors—i.e., $P\ll N_{b}$—requiring the effective recovery dimension *K* to be smaller than the candidate basis dimension $N_{b}$; or (ii) a case in which the candidate basis has no inherent ordering—a key enabler for incrementally better reconstruction. In both situations mentioned above, the algorithm needs to be able to identify the *K*-best coefficients *a* for sparse recovery, which in turn requires sparsity-promoting $l_{1}$ norm minimization reconstruction as given by Equation (6). In this work, we adopt an iterative sequential least-squares thresholding framework to extend the least-squares algorithm used above, and this is presented in Algorithm 2. The idea here is to repeatedly “shrink” the least-squares coefficients using a threshold hyperparameter.

**Algorithm 2:**$l_{1}$-based algorithm: Sparse reconstruction with known basis, Φ. **input**: Full data ensemble $X\in R^{N\times M}$    Incomplete data $\tilde{X}\in R^{N\times M}$    The mask vector $m\in R^{N}$    The chosen sparsity $k_{sparse}$ **output**: Approximated full data $\bar{X}\in R^{N\times M}$

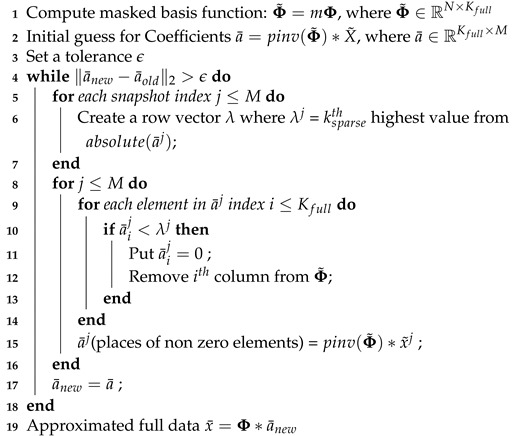



## 5. Algorithmic Complexity

In this brief section, we present the algorithmic complexity of the above methods. Computing the POD basis requires $O(N\times M^{2})$ operations, where N,M are the full state and snapshot dimensions, respectively. The subsequent cost of sparse recovery is O(N×K×M) for both methods, where K≤M is the desired recovery dimension. In practical flows with a low-dimensional structure, POD is expected to result in a smaller *K* than other classes of a data-driven basis. This helps limit the sensor budget and reconstruction cost. Further, since the snapshot dimension (*M*) is tied to the basis dimension (*K*), the larger the *K*, the more snapshots (of dimension *M*) are needed, resulting in a higher computational cost.

The complexity of sensor placement depends on the method chosen. For example, QR factorization with column pivoting requires $O(N^{3})$ operations for an N×N matrix and $O(NM^{2})$ for an N×M matrix. The DEIM method involves a complexity of $O(NM^{3})$ when retaining *M* POD modes and identifying *M* sensors with a full state dimension of *N*. These estimates are consistent with our experience of deploying DEIM and QR-pivoting approaches on the datasets reported in this work.

## 6. Sparse Recovery Use Cases

To demonstrate the performance of sparse recovery using the ELM-GS basis for different sensor placements, we consider two representative flow fields, namely a low-dimensional cylinder wake flow and a more complex geophysical field of sea surface temperature data from NOAA. The SR performance using ELM-GS basis is compared with that of POD-based SR.

### 6.1. Low-Dimensional Cylinder Wake Flow

**Figure 3:**
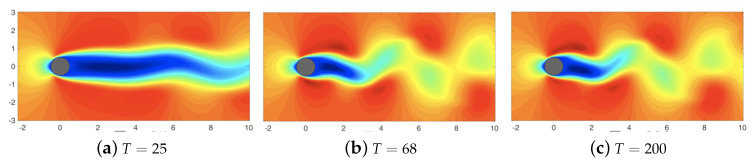
Isocontour plots of the stream-wise velocity component for the cylinder flow at Re=100 at T=25,68,200, showing the evolution of the flow field. Here, *T* represents the time non-dimensionalized by the advection time-scale.

As the first use case, we consider the data-driven sparse reconstruction of the cylinder wake flow fields at a Reynolds number of Re=100 involving unsteady wake dynamics (see Figure 3). The two-dimensional flow data is modeled using a higher-order spectral Galerkin framework [73] *Nektar++* to capture the vortex roll-up process and eddying structure. Specifically, we adopt a fourth order spectral expansion within each element to solve the incompressible Naiver–Stokes equations,

(19a) ∇·u=0,

(19b) $$\frac{\partial u}{\partial t}+u\cdot \nabla u=-\nabla P/\rho +\nu \nabla^{2}u,$$

where *u* and *v* are horizontal and vertical velocity components, *P* is the pressure field and ν is the fluid viscosity. The simulation domain used extends over −25D≤x≤45D and −20D≤y≤20D, where *D* is the diameter of the cylinder. To reduce the state dimension, we consider a reduced domain of extent −2D≤x≤10D and −3D≤y≤3D that encompasses the key flow dynamics. The resulting state dimension is ∼24,000 for each variable, and data snapshots are recorded every Δt=0.2. The mesh distribution ensures that the thin shear layers near the surface are resolved, as is the transient wake physics.

**Figure 4:**
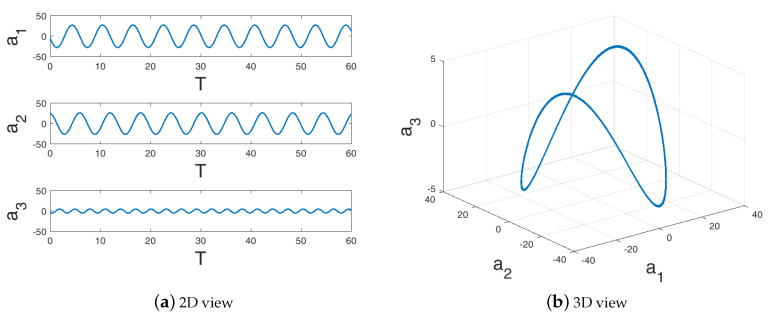
The temporal evolution of the first three normalized proper orthogonal decomposition (POD) coefficients for the limit cycle cylinder flow at Re=100.

**Figure 5:**
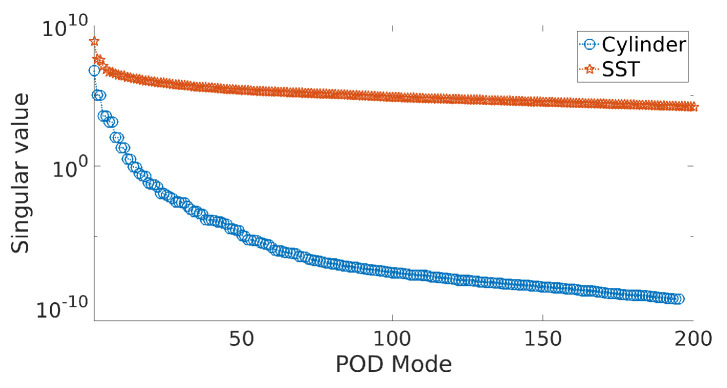
Singular value spectrum of the data matrix for both the cylinder wake flow at Re=100 and the sea surface temperature(SST) data.

The time-evolution of the cylinder wake flow (Figure 3) shows the wake instability and limit-cycle dynamics (Figure 4). The rapid decay of the singular value spectrum (Figure 5) clearly shows that the system evolves in a low-dimensional space. In this study, we use 300 snapshots collected (every 0.2 non-dimensional time units) over 60 non-dimensional times, $T=\frac{Ut}{D}$ which represents ∼10 cycles of the dynamics.

### 6.2. Global Sea Surface Temperature (SST) Data

Representing a more complicated use case for the methods presented in this article, the sea surface temperature (SST) dataset represents synoptic-scale ocean turbulence and is made available by the National Oceanic & Atmospheric Administration (NOAA) (https://www.esrl.noaa.gov/psd/).

**Figure 6:**
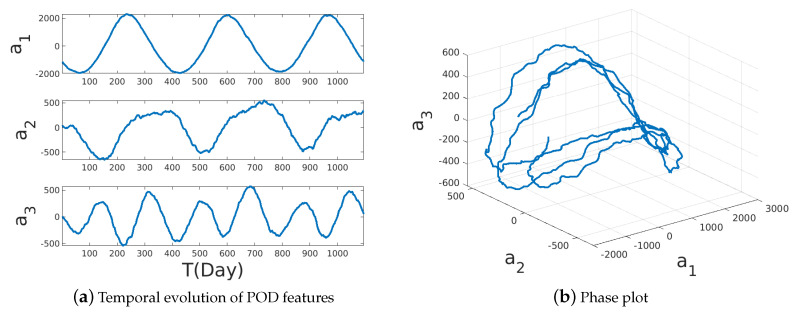
The temporal evolution of the first three normalized POD coefficients for the sea surface temperature (SST) data.

The data represent a filtered turbulent field as they represent the daily mean temperature from high-resolution blended analysis for the year 2018. The dataset includes daily snapshots (for 365 days) of a temperature field with a spatial resolution of 0.25° longitude × 0.25° latitude, resulting in a total state dimension of 720×1440. Of this full state dimension of 1,036,800 observations, only 691,150 (≈69%) measurements correspond to non-landed regions and are used here. The singular value spectra (Figure 5) for this dataset shows a slow decay of eigenvalues as compared to the low-dimensional wake flow and is therefore higher dimensional. In spite of the turbulent nature of this data, the dynamics of the POD features in Figure 6 show nearly periodic evolution at the large scales.

## 7. Assessment of Dimensionality, Basis Structure and Hierarchy for Sparse Recovery

### 7.1. Dimensionality

Data-driven bases vary in their capacity to represent full state information as quantified through the number of basis vectors of a given basis set to represent the full state up to a desired accuracy; i.e., the system dimensionality in a given basis space. For a POD basis that is energy-optimal, the knowledge of the singular value spectrum (Equation (8)) precisely informs us of the energy content in each mode and also allows for characterization of the cumulative energy, $E_{K}=\sum_{k=1}^{K}\frac{\lambda_{k}}{\left(\lambda_{1}+\lambda_{2}+\cdots +\lambda_{M}\right)}\times 100$ as retained in the reconstruction up to a desired mode *K*. For the low-dimensional limit cycle wake dynamics at Re=100, two and five POD modes (of the 300 basis vectors computed) are required to capture 95% and 99% of the energy content (variance), respectively. We also compute $err_{K}^{POD}={\left|\left|X-\Phi_{K\times N}^{POD}a_{K\times M}^{POD}\right|\right|}_{2}$, where $\Phi_{K\times N}^{POD},\;a_{K\times M}^{POD}$ represents the matrix comprising *K* POD vectors and the corresponding coefficients for the different snapshots, respectively. Relating the system dimension with energy from the singular value spectrum and reconstruction error offers a way to compare different bases that may be “ordered” and “unordered” in some way.

**Figure 7:**
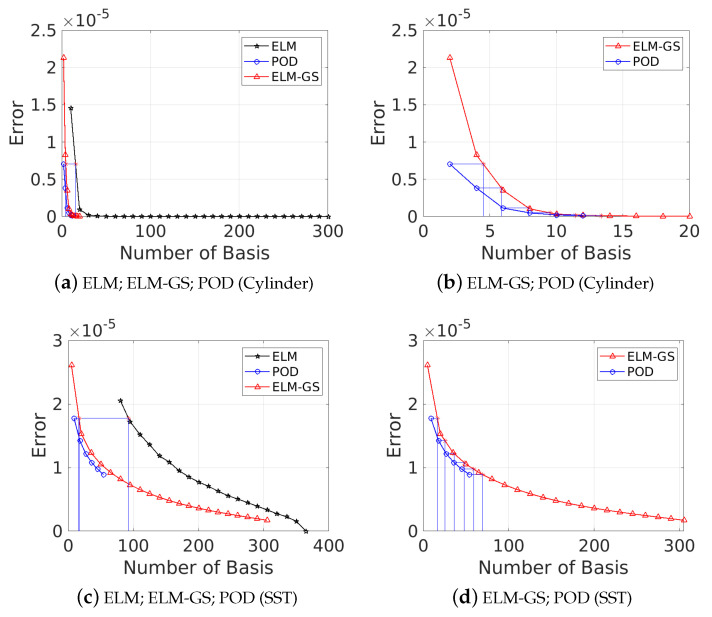
Reconstruction error ($err_{K}^{POD},err_{K}^{ELM},err_{K}^{ELM-GS}$) decay using different numbers of bases (*K*) for POD, ELM and Gram–Schmidt extreme learning machine (ELM-GS) bases, considering both cylinder wake data (top row) and sea surface temperature (SST) data (bottom row).

In such situations, characterizing the system dimension *K* through the reconstruction error (with respect to the true data) offers a way forward. For example, in the case of ELM, the training error from the ELM network (Equation (14)) may be used. The error is quantified according to the Frobenius norms denoted by $err_{K}^{ELM}={\left|\left|X-X_{K}^{ELM}\right|\right|}_{2}$ and $err_{K}^{ELM-GS}={\left|\left|X-X_{K}^{ELM-GS}\right|\right|}_{2}$ using the *K*-modal reconstruction of the flow fields, $X_{K}^{ELM}=\Phi_{train,K\times N}^{ELM}a_{train,K\times M}^{ELM}=\Phi_{K\times N}^{ELM}a_{K\times M}^{ELM}$ and $X_{K}^{ELM-GS}=\Phi_{train,K\times N}^{ELM-GS}a_{train,K\times M}^{ELM-GS}=\Phi_{K\times N}^{ELM-GS}a_{K\times M}^{ELM-GS}$, respectively. A simple method of estimating the system dimension in any basis is to compare the reconstruction error with the corresponding POD-based reconstruction which optimally captures the variance in the data. Figure 7 shows the comparison of the decay of representation errors with dimension for the different bases, and Table 1 quantifies the dimension corresponding to 95% and 99% energy in terms of POD singular value spectra. We clearly see from Figure 7a that the POD basis offers the most parsimonious representation of the data ($K_{95}=2,K_{99}=5$), followed by ELM-GS ($K_{95}=6,K_{99}=7$) and ELM ($K_{95}=16,K_{99}=19$). The corresponding values for the high-dimensional SST data are also tabulated. The ELM-GS is only slightly more expensive than POD (Figure 7b), although it spans nearly the same subspace as the ELM basis.

### 7.2. Basis Structure

**Figure 8:**
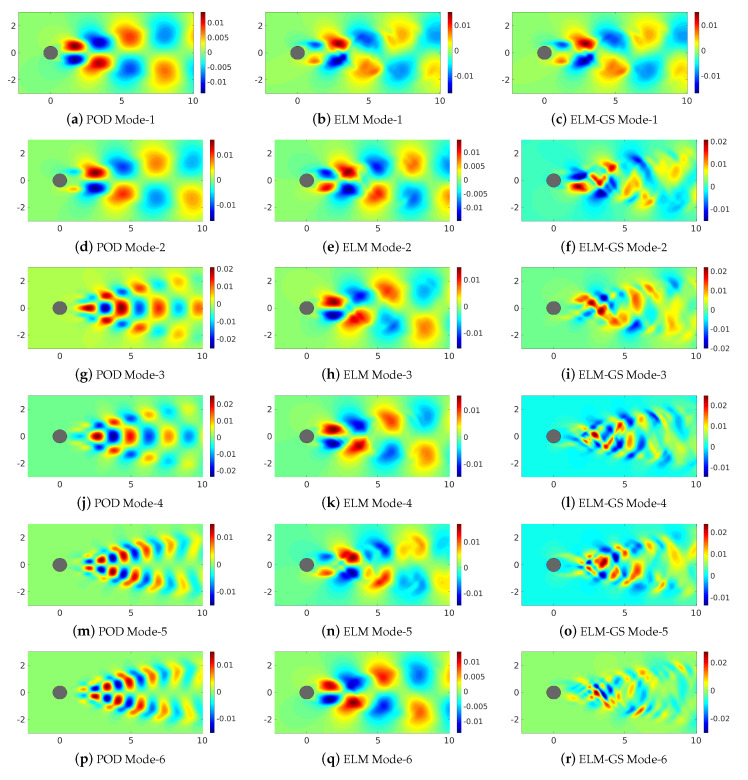
Comparison of the first six modes of POD, ELM and ELM-GS. The POD and ELM-GS share similar structures, possibly due to their underlying orthogonality, while ELM represents repeating structures not unlike POD modes 1 and 2.

**Figure 9:**
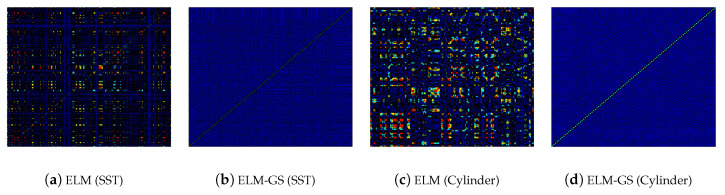
$\Phi^{T}\Phi$ contour plot of ELM and ELM-GS basis for both cylinder wake and sea surface temperature (SST) data. Red indicates a value of one, and blue indicates a value of zero.

Having compared the dimension of the data in different basis subspaces, we also look at the topology of the basis vectors. In Figure 8, we compare the first six modes for the POD, ELM and ELM-GS for the cylinder wake flow. The well-known orthogonal structure of the POD basis for the cylinder wake contrasts with the qualitative similar structure of the ELM modes (modes 1–3 and 5–6 are similar to each other), while that of the ELM-GS displays a tendency to transition from the ELM modal structure to the orthogonal POD modal structure with increasingly smaller eddies at the higher modes. This semblance of scale hierarchy of the ELM-GS modes contributes to their ability to accurately represent data using fewer modes. We quantify the basis orthogonality using the product $\Phi^{T}\Phi$ for both ELM and ELM-GS in Figure 9. These plots show clear diagonal dominance for the ELM-GS basis.

### 7.3. Basis Hierarchy

**Figure 10:**
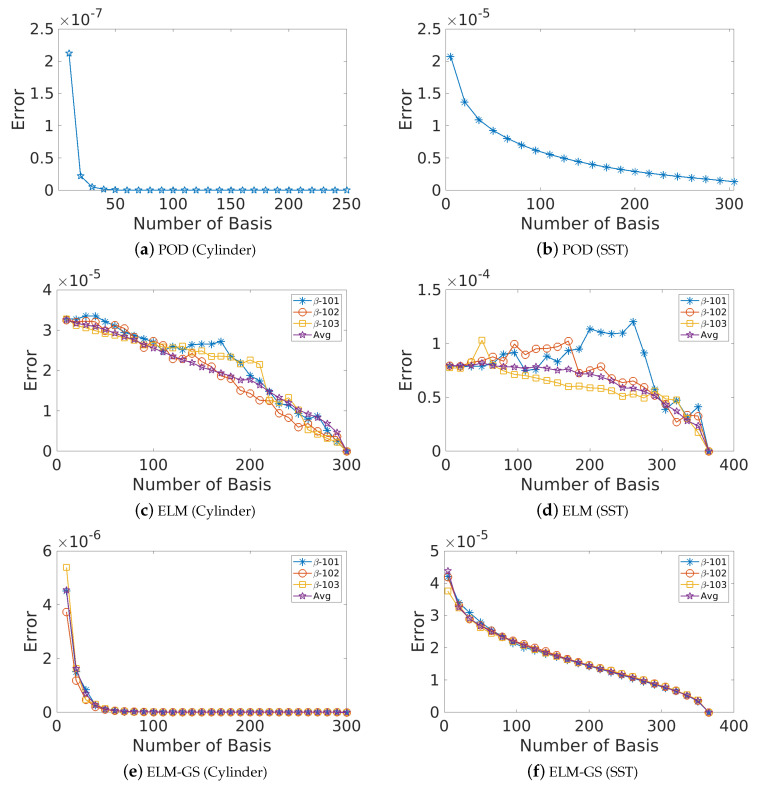
Plots showing how the error decays with the increase of basis number for all three cases POD, ELM and ELM-GS for both cylinder wake data (left column) and sea surface temperature (SST) data (right column). For ELM and ELM-GS, the different realizations corresponding to the different random seeds used for the network weights in the ELM (SLFN) training are shown, as well as the average error over 20 different training samples.

For a given dataset, the generated POD modes offer built-in ordering; i.e., one can sequentially include more modes to generate increasingly accurate representations of the true data. This is not likely the case for non-optimal basis choices such as Fourier or ELM bases. Here, we explore this aspect of the basis hierarchy for ELM and ELM-GS bases in comparison to that of POD modes by incrementally adding basis vectors to recover the flow field while tracking the error decrease in the reconstructed field. Outcomes from this analysis are presented in Figure 10 for both the chosen datasets. We clearly observe that both ELM-GS and POD show a systematic decrease of the reconstruction error with an increase in the number of basis vectors, *K*, and the error decay is rapid for low-dimensional reconstruction; in contrast, for the ELM basis, we clearly see a non-monotonic error decay, although the overall trend shows an error decrease as expected. These trends are verified for the multiple choice random initialization of the weights in the ELM training, as denoted by the seed β. The outcomes clearly show that ELM-GS introduces a consistent basis hierarchy independent of the ELM training and is therefore a robust choice for sparse recovery applications.

## 8. Assessment of Sparse Reconstruction Performance 

### 8.1. Sparse Reconstruction Experiments, Analysis Methods and Error Quantifications

Having explored the ability of the different basis spaces to approximate the data, we now assess their linear sparse estimation performance using multiple sensor placement strategies. To accomplish this, we reconstruct the full field from sparse data using numerically simulated flow fields and observation datasets (NOAA-SST). In the offline stage, the full field representation is used to learn the data-driven basis and sensor locations. In practice, the sensor locations are identified using prior knowledge of the system. The concept of data-driven sensor placement is adopted here with the aim of identifying choices that provide robust outcomes with accuracy. In this study, we design sensors as fixed (in time) single-point measurements using random or smart sampling algorithms such as DEIM or QR-factorization with column pivoting. This offline step yields at most *M* bases (*M* is the number of snapshots) for use in the reconstruction process in Equation (2) (candidate basis dimension of $N_{b}=M$) and *P* (desired) sensor locations. The desired recovery dimension *K* can be chosen as $N_{b}$ or smaller ($K<N_{b}$). The earlier discussion from Section 2 and prior studies [48] shows us that, for a chosen *K*, P≥K is likely to generate reasonable results using $l_{2}$ reconstruction with $K=N_{b}$. If $N_{b}$ is large, the best subset of *K* bases is generally chosen to generate an accurate reconstruction by looking for a *K*-sparse solution using $l_{1}$ methods. If the basis vectors are ordered in terms of their “relevance” to this dataset, then the best subset of *K*-bases will also be the first *K*-bases of the sequence. We use this as a way to verify the basis hierarchy in POD and ELM-GS by comparing the outcomes from $l_{1}$ (with $M=N_{b}>K$) and $l_{2}$ (with $N_{b}=K$) methods. Once the basis hierarchy is established, we evaluate the reconstruction performance by comparing the true flow field with those from SR using POD and ELM-GS bases for an ensemble of numerical experiments spread over different sensor budgets, *P*, and reconstructed system dimensions, *K*, using $l_{2}$ methods.

Assessing the accuracy of the sparse recovery outcomes across a wide range of design parameters is challenging. For example, two POD modes may generate the same reconstruction accuracy as five ELM-GS modes, as shown in Table 1. Further, two different flows may have different scale separations and therefore dimensionality in a basis space. To address this, we first define the various normalized metrics for the comparison and generalization of outcomes as used in our earlier work [47,48]. We recount these briefly for completeness.

To illustrate these ideas, we note that two POD modes capture 95% of the energy for the cylinder wake flow ($K_{95}^{POD}=2$) while the SST data require nine modes ($K_{95}^{POD}=9$). Therefore, analysis across different flow regimes and algorithms requires thenormalization of the system dimension as $K^{*}=K/K_{95}$ and a normalized sensor budget, $P^{*}=P/K_{95}$, to be handled. Using this, we design an ensemble of sparse recovery experiments in the normalized $P^{*}-K^{*}$ space over the range $1<K^{*}<6$ and $1<P^{*}<12$ for the different choices of bases and sensor placements. The lower bound of one aspect is chosen so that the minimally accurate reconstruction captures 95% of the energy—this choice is left to the user. To quantify the flow field recovery performance for the different problem designs, we define the mean squared reconstruction error as

(20) $$\epsilon_{K^{*},P^{*}}^{SR}=\frac{1}{M}\frac{1}{N}\sum_{j=1}^{M}\sum_{i=1}^{N}{\left(X_{i,j}-{\bar{X}}_{i,j}^{SR}\right)}^{2},$$

where *X* is the true data and ${\bar{X}}^{SR}$ is the recovered field using sparse measurements; *N* and *M* represent the state and snapshot dimensions affiliated with indices *i* and *j*, respectively. We also define the mean squared errors $\epsilon_{K_{95}^{*}}^{FR}$ and $\epsilon_{K^{*}}^{FR}$ for the full reconstruction (FR) using the different bases; namely, POD and ELM-based SR are

(21) $$\epsilon_{K_{95}^{*}}^{FR}=\frac{1}{M}\frac{1}{N}\sum_{j=1}^{M}\sum_{i=1}^{N}{\left(X_{i,j}-{\bar{X}}_{i,j}^{FR,K_{95}^{*}}\right)}^{2};\;\epsilon_{K^{*}}^{FR}=\frac{1}{M}\frac{1}{N}\sum_{j=1}^{M}\sum_{i=1}^{N}{\left(X_{i,j}-{\bar{X}}_{i,j}^{FR,K^{*}}\right)}^{2},$$

where ${\bar{X}}^{FR}$ is the reconstruction using exact coefficients for the different bases, $K_{95}^{*}=K_{95}/K_{95}=1$ is the normalized system dimension corresponding to 95% energy capture and $K^{*}=K/K_{95}$ represents the desired reconstructed system dimension. Therefore, the FR errors represent the best case values; i.e., lower bounds for the sparse recovery errors. This enables us to define normalized error metrics representing the absolute ($\epsilon_{1}$) and relative ($\epsilon_{2}$) measures as

(22) $$\epsilon_{1}=\frac{\epsilon_{K^{*},P^{*}}^{SR}}{\epsilon_{K_{95}^{*}}^{FR}}\;,\;\epsilon_{2}=\frac{\epsilon_{K^{*},P^{*}}^{SR}}{\epsilon_{K^{*}}^{FR}}.$$

These normalized metrics allow us to compare both the “absolute” and relative reconstruction quality for a given problem design (i.e., P,K). While $\epsilon_{1}$ represents the SR error normalized by the corresponding full reconstruction error for 95% energy capture, $\epsilon_{2}$ represents the relative SR performance obtained by normalizing the SR error with the FR error for the desired reconstruction accuracy (for dimension *K*). These normalized metrics enable us to compare the different SR algorithms/design choices across different flow regimes.

### 8.2. Basis Hierarchy in ELM-GS and POD Bases

**Figure 11:**
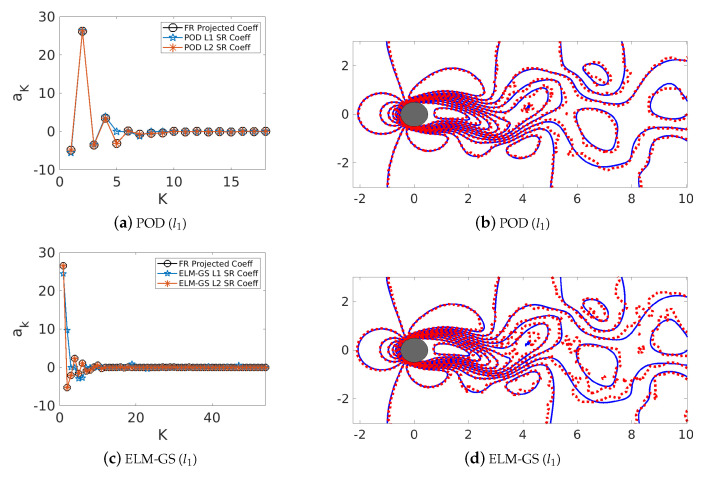
Normalized projected and reconstructed coefficient *a* (**a**,**c**) and The line contour comparison of the streamwise velocity between the actual CFD solution field (blue) and the energy-based SR reconstruction (red) using the $l_{1}$ SR algorithm for both POD and ELM-GS-based reconstruction (**b**,**d**) at $K^{*}=9,P^{*}=18$.

We have shown through the decay of reconstruction errors in Section 7.3 that POD and ELM-GS bases have inherent hierarchical structures for flow recovery. Here, we establish the same by comparing a *K*-sparse recovery of high-resolution data from heavily downsampled data using both $l_{2}$ and $l_{1}$ minimization approaches. For these experiments using the cylinder wake flow data, we build a candidate basis library of dimension 200 from which the desired sparse solution is estimated using DEIM-based sparse measurements. DEIM-based sensing is attractive due to its computational efficiency and the ability to identify physically relevant sensor locations. In Figure 11, we compare the reconstructed instantaneous flow field and estimated sparse coefficients with the corresponding ground truth for a case with $P^{*}=18,K^{*}=9$. For both the POD and ELM-GS basis, we see that $l_{1}$ minimization using the sequential thresholded least squares (Section 4.1) excite only the first few coefficients (Figure 11a,c), similar to $l_{2}$ minimization, thereby verifying that the bases are ordered in terms of their relevance to the data. Leveraging this outcome, we pursue the rest of this analysis using least-squares minimization methods.

### 8.3. Comparison of Sensor Placement Using ELM-GS and POD Bases

**Figure 12:**
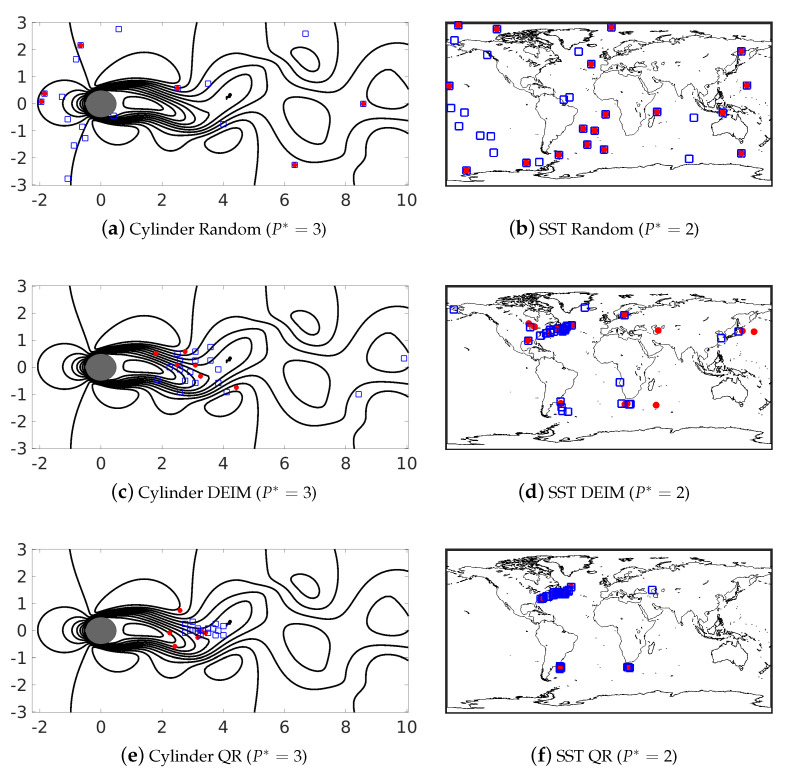
Sensor locations chosen using random (**top row**), discrete empirical interpolation method (DEIM) (**middle row**) and QR-pivoting (**bottom row**) sensor placement methods for budgets of $P^{*}=3$ for cylinder flow (left) and $P^{*}=2$ for SST data (right). Red dots: POD-basis-based sensor location; blue square: ELM-GS-basis-based sensor locations.

Using knowledge of the underlying data-driven bases, data/physics-informed sensor placement can be determined, as discussed in Section 3; this can in turn be used for sparse recovery. In this study, we use both ELM-GS and POD bases to identify smart sensors, as shown in Figure 12 for the cylinder wake flow and SST data. The red dots in the plots are generated using the POD-basis, while the blue dots are estimated using ELM-GS. We also include the random sensor placement for comparison purposes. The different columns in each of these figures correspond to different normalized sensor budgets, $P^{*}$. As ELM-GS is slightly higher dimensional than POD, we see more blue squares in the figures than red dots. Unlike random sensor placement, the physics-informed sensor placement methods—namely DEIM and QR pivoting—generate sensors in the dynamically relevant regions of the flow; specifically, the wake region of the cylinder and the coastal regions for the SST data in Figure 12. That said, both ELM-GS and POD-based sensors identify hardly any of the same locations for both of these different flow patterns. The overlap in sensor locations observed for random placement is due to the algorithm sampling the raw full state data.

### 8.4. Sparse Recovery Error Dependence on Sensor Budget and System Dimension using ELM-GS and POD Bases

**Figure 13:**
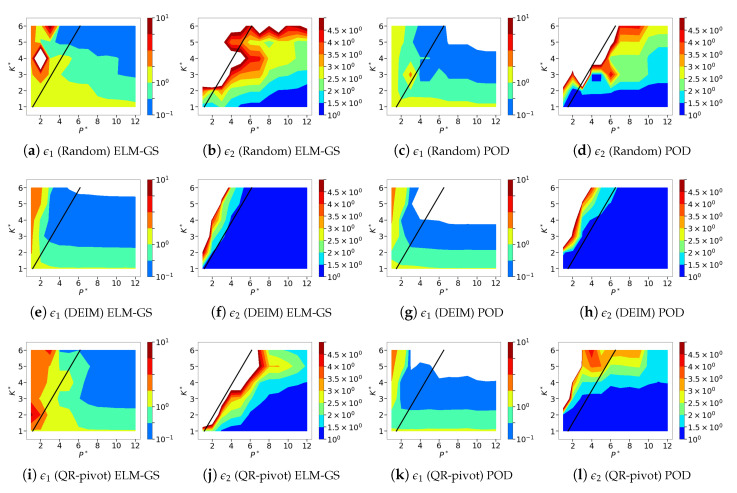
Isocontours of the normalized mean squared ELM-GS (**a**,**b**,**e**,**f**,**i**,**j**) and POD-based (**c**,**d**,**g**,**h**,**k**,**l**) sparse reconstruction errors ($l_{2}$ norms) using DEIM (top row), QR-pivoting (middle row) and random (bottom row) sensor placements for cylinder wake data. Left: normalized absolute error metric, $\epsilon_{1}$. Right: normalized relative error metric, $\epsilon_{2}$. The black line corresponds to $P^{*}=K^{*}$ and separates the over-sampled form under-sampled regions.

**Figure 14:**
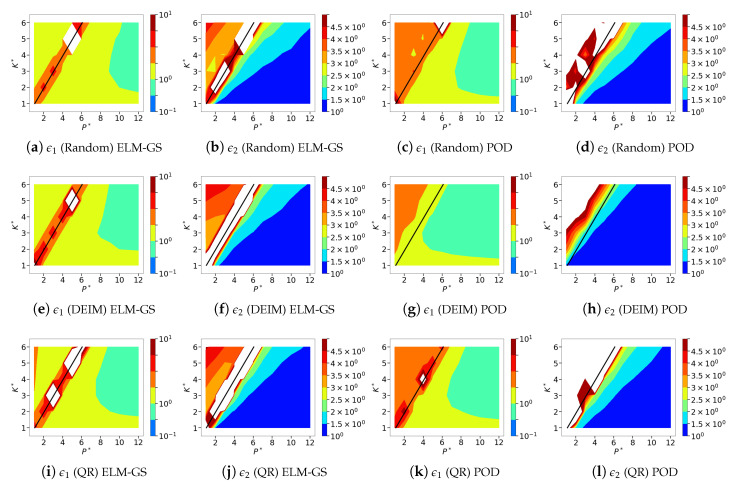
Isocontours of the normalized mean squared ELM-GS (**a**,**b**,**e**,**f**,**i**,**j**) and POD-based (**c**,**d**,**g**,**h**,**k**,**l**) sparse reconstruction errors ($l_{2}$ norms) using random (top row), DEIM (middle row) and QR-pivoting (bottom row) sensor placements for sea surface temperature (SST) data. Left: normalized absolute error metric, $\epsilon_{1}$. Right: normalized relative error metric, $\epsilon_{2}$. The black line corresponds to $P^{*}=K^{*}$ and separates the over-sampled form under-sampled regions.

In this section, we analyze the sparse recovery performance using the different sensor placements and basis choices over the parameter space of the normalized sensor budget $P^{*}$ and system dimension $K^{*}$ for both classes of fluid flows. We refer the reader to Section 8.1 for the experimental details and definition of the error metrics. Given that the parameter space for our analysis is four-dimensional, we focus only on the major conclusions instead and limit in-depth analysis using instantaneous flow fields to a few instances. In Figure 13, we present 12 different isocontour plots of the normalized error metrics $\epsilon_{1}$ and $\epsilon_{2}$ (see Equations (21) and (22)) corresponding to three different sensor placement strategies and two different bases for the sparse recovery of the cylinder wake flow. The corresponding figure for the SST data is presented in Figure 14. In general, the smaller the sensor budget, the more sensitive the SR is to bad sensor placement. This observation tends to be applied to highly parsimonious low-dimensional bases such as POD as compared to less parsimonious bases such as ELM [47,48]. In this study, we assess how orthogonalized ELM-GS bases fare with respect to the different sensor placement methods. As mentioned above, $\epsilon_{1}$ represents an absolute normalized error; i.e., an SR error in the estimated flow field normalized by an error quantity that is specific to the given flow, whereas $\epsilon_{2}$ represents a relative normalized error using normalization by an error metric not only specific to the flow, but also to that particular dimension, *K*, up to which the system recovery is sought. Therefore, one can see that as the sensor budget *P* and the targeted recovery dimension *K* increase, $\epsilon_{1}$ should decrease, but only up to the corresponding full data reconstruction error, $\epsilon_{K^{*}}^{FR}$. Consequently, $\epsilon_{2}$ should asymptote to a value of unity at a large enough $P^{*}$.

Against this background, we now evaluate the SR performance over the entire parametric design space. We see that for the all the different sensor placements and basis choices, the $\epsilon_{1}$ contours in Figure 13 and Figure 14 mostly display characteristic L-shaped contour variations in line with the expected decay of error metrics at higher *P* and *K* values. Similarly, the $\epsilon_{2}$ contours in general tend to approach values of unity for $P^{*}>K^{*}$. The impact of the sensor placements and basis choices is particularly clear in the finer details. In particular, we focus on the marginally over-sampled region; that is, the region where $P^{*}≳K^{*}$. This is motivated by the fact that all the different sampling strategies work favorably in the highly over-sampled regime with $P^{*}\gg K^{*}$. Similarly, in the highly under-sampled limit with $P^{*}<K^{*}$, the linear estimation problem is ill-posed, which produces sparse recovery errors irrespective of the choice of sensor placement. Therefore, a sensitivity to choices of basis and sensor placement is naturally observed in the marginally over-sampled region. In this part of the design space, we observe that both ELM-GS and POD bases show higher errors for QR-pivoting and random sensor placement, while DEIM generates smaller errors. Additionally, DEIM shows fast error decay with an increase in $P^{*}$ as compared to QR-pivoting and random sensor placement. Comparing ELM-GS and POD, we see that ELM-GS shows higher errors and slower decay rates with an increase in $P^{*}$ compared to the POD-basis for both random sensors and QR-pivoting. Overall, both sets of bases show higher errors when using these sensor placement strategies for the low-dimensional cylinder wake while their performance is relatively accurate when using DEIM.

For the higher-dimensional NOAA-SST dataset, both ELM-GS and POD show reasonable SR accuracy when using both QR-pivoting and random sensing. However, ELM-GS shows slightly faster error ($\epsilon_{2}$) decay with $P^{*}$ compared to POD-based SR when using QR-based sensors. With DEIM sensor placement, ELM-GS shows reasonable SR accuracy, but error $\epsilon_{2}$ decays slowly with $P^{*}$ as compared to POD-based SR, which shows very high levels of accuracy. Overall, for these high-dimensional data, ELM-GS offers consistent performance across the different sensor arrangements including QR-pivoting, while POD-based SR shows clear benefits from DEIM.

**Figure 15:**
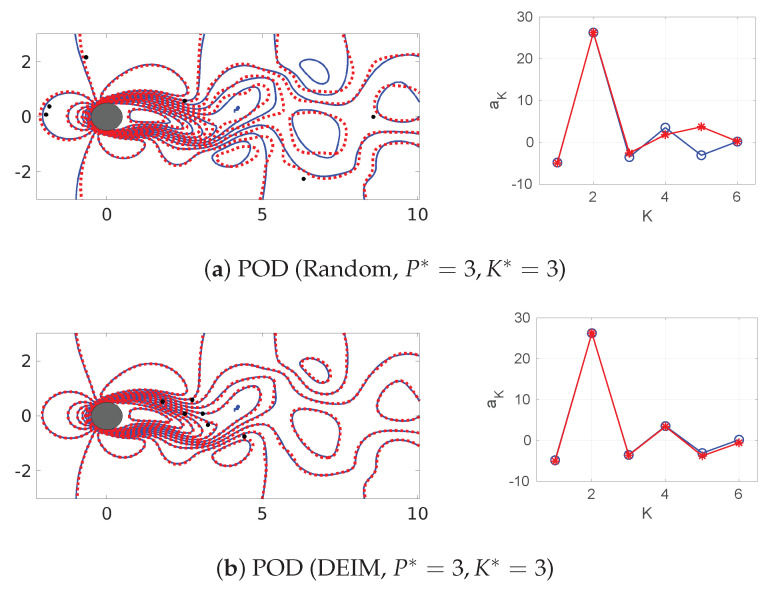

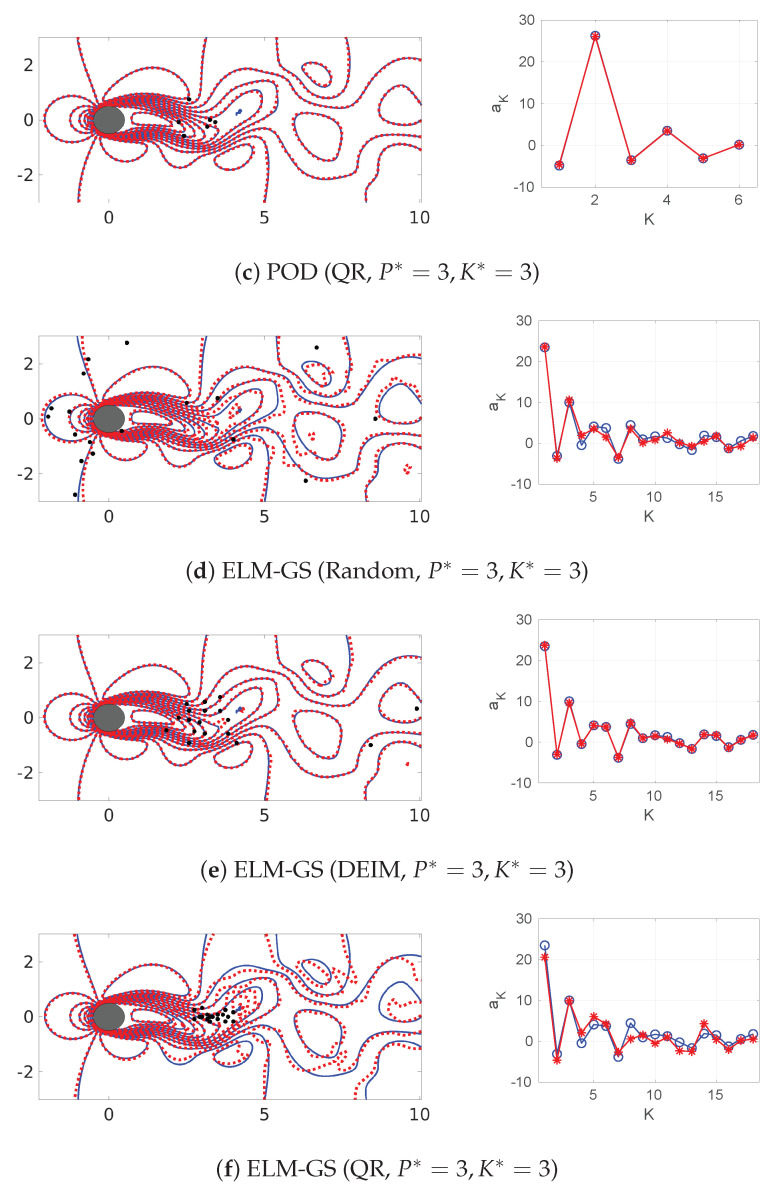
Left column: Comparison of line contours of streamwise velocity between the true flow field (blue) and SR reconstruction (red) for Re=100 using random and DEIM sensor placement at $P^{*}=3,K^{*}=3$ (marginally sampled) using both ELM-GS and POD SR. Right column: comparison of the estimated coefficients *a* using the entire data (blue circle) and the downsampled data (red star).

We further examine the above results to interpret the observed trends through instantaneous isocontours and basis coefficients/features estimated from sparse recovery. Since we have performed more than a thousand sparse recovery computations for this analysis, we selectively analyze the cases chosen for this dissection step. As marginal oversampling with $P^{*}⪆K^{*}$ displays the most variability in the averaged error metrics across the different design choices (in Figure 13 and Figure 14), especially in the vicinity of the $P^{*}=K^{*}$ line (in black in the figures), we focus on cases where $P^{*}\approx K^{*}$. In the left column of Figure 15, we exaime the reconstructed flow field by comparing the sparse recovered field with the exact full-resolution structure of dimension $K^{*}$ for the two basis choices and sensor placements at $P^{*}=K^{*}=3$; i.e., for a marginally sampled flow. In addition, we also compare the POD/ELM-GS basis features recovered by the SR algorithm with the exact values as shown in the right column of Figure 15. We clearly observe that, for the POD-based SR, the snapshot reconstruction with random sensors shows the most error for this low-dimensional wake flow (Figure Figure 15a) while DEIM and QR-pivoting (Figure 15b,c) show accurate reconstruction. In comparison, the ELM-GS basis requires a slightly higher number of sensors (denoted by black dots in the isocontour plots) and performs accurately using DEIM sensors (Figure 15e) followed by random sensor placement (Figure 15d) and QR-pivoting-based sensor placement in Figure 13f with the most error. Although the dissection of single snapshot reconstructions such as this may not capture all the trends in the averaged error metrics shown in Figure 13, we observe the following dominant trends from visual inspection: (i) DEIM sensor placement offers better performance and (ii) the ELM-GS basis paired with random/QR-pivoting sensors generates higher errors compared to the corresponding POD-based SR.

**Figure 16:**
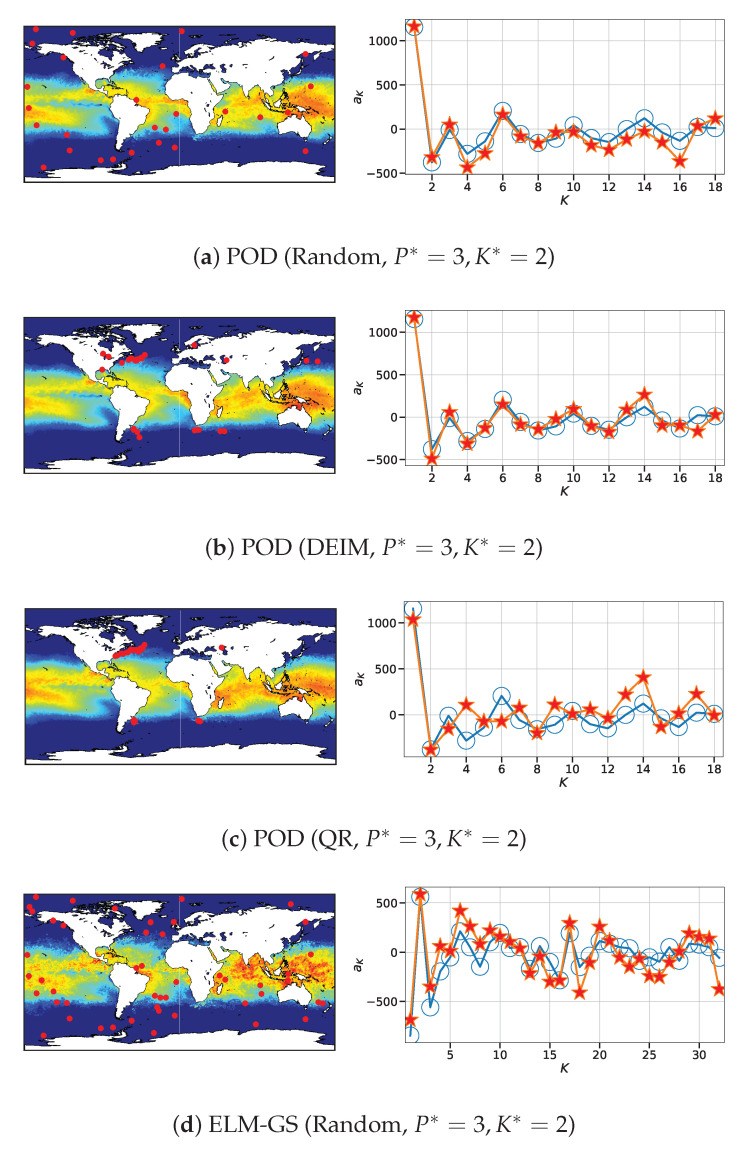

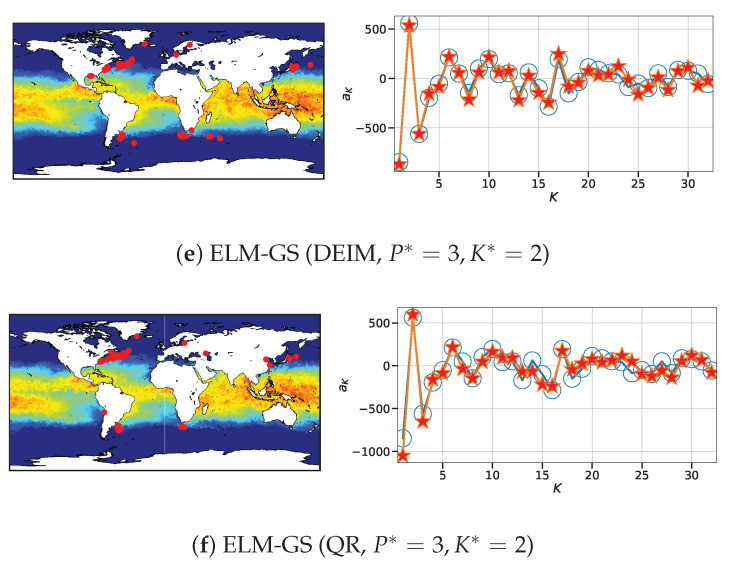
Left column: SR plot for sea surface temperature (SST) data with POD and ELM-GS basis using random, DEIM and QR sensor placement sampled marginally ($P^{*}=3,K^{*}=2$). Right column: comparison of the estimated coefficients *a* using the entire data (blue circle) and the downsampled data (red star). Contour color: dark blue represents a temperature equal to or below 15° celsius and red represents a temperature equal or above 35° celsius.

For the high-dimensional SST, we examine the instantaneous flow field and basis features estimated for the marginally oversampled case $P^{*}=3$ and $K^{*}=2$ in Figure 16. In particular, we note that POD-based SR (Figure 16a–c) shows lower errors in the estimation of the basis features for DEIM with a relative degradation in performance for both random and QR-pivoting-based sensors, increasing in that order. The ELM-GS counterpart in Figure 16d–f shows larger deviations from the ground truth for random sensing while showing improved accuracy for DEIM and QR pivoting. Once again, the trends from single snapshot reconstruction dissection of the NOAA-SST data are consistent with those gleaned from the averaged error metrics in Figure 14; in particular, it is shown that ELM-GS performs better with QR-pivoting for this dataset as compared to POD-based SR. The superior performance of DEIM for both these use cases is not surprising as the sensor placement algorithm directly leverages knowledge of the basis vectors used in the SR step. However, the performance for both POD and ELM-GS-based SR with QR-pivoting generates results that are problem-dependent. To investigate this, we inspect the matrix condition numbers below.

A key metric that impacts SR performance in linear estimation methods is the condition number of the matrix, θ. Consistent with the least-squares minimization algorithm used in this work, we explore the condition number for $\Theta^{T}\Theta$ in Table 2 for different bases, $P^{*}-K^{*}$ combinations and sensor placement methods for the reconstruction of the NOAA-SST dataset.

We clearly see that POD-based SR shows smaller (O(100)) condition numbers for DEIM sensor placement, even for the marginally sampled cases. For QR-pivoting and random sensing, we see that significant oversampling with $P^{*}=2K^{*}$ is needed to ensure the condition number drops to reasonable values. In comparison, ELM-GS shows larger condition numbers than POD-based SR on average, but it is more sensitive to sensor budgets than sensor placement; that is, higher sensor budgets in the marginally oversampled and oversampled limits result in smaller condition numbers, even for random and QR-pivoting-based sensing. This is in contrast to POD-based SR, which shows large condition numbers for similar SR designs. In summary, this analysis confirms that SR performance improves with oversampling and sensor placement, which is tied to the data. While POD-based SR responds better to high-quality data-informed sensor placement methods such as DEIM, ELM-GS responds better to oversampling even with random and less-than-ideal sensing strategies. This explains the better SR accuracy generated for the NOAA-SST dataset using ELM-GS with QR-pivoting in the marginally sampled limit as compared to POD-based SR.

## 9. Discussion and Conclusions

In this work, we have presented a framework for data-driven sensor placement and sparse reconstruction using arbitrary non-orthogonal bases that may be encountered in a machine learning workflow to handle complex dynamical systems. Although this work has adopted projections using ELM autoencoder maps for the low-dimensional representation of the data, the methods presented here can, in principle, be applied to any arbitrary class of basis vectors. Naturally, the success of the procedure depends on the effectiveness of the basis vectors in approximating the space described by the data. In addition to the lack of parsimony, arbitrary non-orthogonal basis tend to suffer from ineffective sensor placement and high algorithmic complexity. In this study, we pair the ELM-basis, which suffers from these deficiencies, with a Gram–Schmidt orthogonalization step to build an ELM-GS basis space as a mitigation step. We compare the basis structure, data-driven sensing and sparse recovery performance of ELM-GS with that using the POD basis.

We observe a reduction of nearly an order of magnitude in the basis dimension for ELM-GS to achieve desired data reconstruction accuracy, which in turn allows for a substantial reduction in sensor requirements for sparse recovery. In fact, most linear estimation algorithms require a sensor budget P≳cK, where *K* is the desired recovery dimension and *c* is a pre-constant; that is, O(1−10). The larger the *K*, the larger the sensor budget *P*. This relationship between *P* and *K* has been verified in our earlier work [48] and also confirmed in this study for the ELM-GS basis in Section 8.4. In fact, our analysis shows that the pre-constant *c* for the ELM-GS basis is ≈1.5. In addition, the topology of the orthogonal ELM-GS modes mimics that of the POD modes for the same data. Therefore, the resulting data-driven sensor placements for both POD and ELM-GS bases show a significant overlap of locations, as reported in Section 8.3. Further, the ELM-GS basis also possesses a built-in hierarchy similar to the POD basis—a trait useful in sparse recovery applications. This allows us to adopt computationally efficient least-squares minimization algorithms to solve the linear estimation problem instead of a more expensive convex optimization problem in a $l_{1}$ formulation.

Reconstructing low-dimensional flows from sparse data, we observe that both POD and ELM-GS-based methods generate similar trends, with DEIM-based sensors showing the highest accuracy followed by QR-pivoting and random sensing. On average, ELM-GS-based SR generates slightly higher errors and slower error decay within the sensor budget *P* in a marginally oversampled regime for both classes of flows considered in the work. However, exceptions do exist, especially when recovering high-dimensional systems such as the SST fields where the different linear estimation methods show reduced accuracy. This is an expected consequence of dealing with multiscale systems, as most sparse estimation methods tend to do well in capturing the larger-scale dynamics but do not work as well at smaller scales. We note that POD-based SR responds better to improved sensor placement from DEIM, while ELM-GS-based SR responds more to slight oversampling, even with less-than-ideal sensor placement, such as by using random sensors. This robustness of ELM-GS to sensor placement is valuable in practical settings where sensors are often distributed randomly.

## Figures and Tables

**Table 1:** Dimension estimation ($K_{95}$ and $K_{99}$) for POD, ELM and ELM-GS corresponding to 95% and 99% energy using a POD reconstruction for both cylinder wake and sea surface temperature (SST) data.

Case		Basis		
		POD	ELM	ELM-GS
Cylinder	$K_{95}$	2	16	5
	$K_{99}$	5	19	7
SST	$K_{95}$	9	92	16
	$K_{99}$	66	195	83

**Table 2:** Condition number estimation of $\Theta^{T}\Theta$ for both POD and ELM-GS basis-based SR using different sensor placement methods on sea surface temperature (SST) data. We have bolded the metrics smaller than a cutoff of 200 to highlight the low condition number cases.

Data: SST		Random	QR	DEIM
POD	Marginally sampled (K* = 2, P* = 2)	2.95 × 10${}^{5}$	$1.21\times 10^{4}$	**35.30**
	Marginally sampled (K* = 2, P* = 2 (+2))	$2.24\times 10^{3}$	$1.28\times 10^{3}$	**35.96**
	Marginally oversampled (K* = 2, P* = 3)	$2.19\times 10^{2}$	$1.22\times 10^{2}$	**30.52**
	Oversampled (K* = 2, P* = 4)	**66.98**	**36.84**	**23.64**
ELM-GS	Marginally sampled (K* = 2, P* = 2)	$5.87\times 10^{5}$	$8.99\times 10^{3}$	$1.72\times 10^{4}$
	Marginally sampled (K* = 2, P* = 2 (+3))	$9.28\times 10^{3}$	$1.22\times 10^{3}$	$1.35\times 10^{3}$
	Marginally oversampled (K* = 2, P* = 3)	**197.18**	**74.78**	**74.42**
	Oversampled (K* = 2, P* = 4)	**45.60**	**33.33**	**29.96**

## References

[1] Jayaraman B., Brasseur J. (2018). Transition in Atmospheric Boundary Layer Turbulence Structure from Neutral to Moderately Convective Stability States and Implications to Large-scale Rolls. arXiv.

[2] Jayaraman B., Brasseur J. Transition in atmospheric turbulence structure from neutral to convective stability states. Proceedings of the 32nd ASME Wind Energy Symposium.

[3] Davoudi B., Taheri E., Duraisamy K., Jayaraman B., Kolmanovsky I. (2020). Quad-rotor flight simulation in realistic atmospheric conditions. AIAA J..

[4] Allison S., Bai H., Jayaraman B. Modeling trajectory performance of quadrotors under wind disturbances. Proceedings of the 2018 AIAA Information Systems-AIAA Infotech@ Aerospace Meeting.

[5] Allison S., Bai H., Jayaraman B. (2020). Wind estimation using quadcopter motion: A machine learning approach. Aerosp. Sci. Technol..

[6] Allison S., Bai H., Jayaraman B. Estimating wind velocity with a neural network using quadcopter trajectories. Proceedings of the AIAA Scitech 2019 Forum.

[7] Jayaraman B., Allison S., Bai H. Estimation of Atmospheric Boundary Layer Turbulence Structure using Modelled Small UAS Dynamics within LES. Proceedings of the AIAA Scitech 2019 Forum.

[8] Holmes P., Lumley J.L., Berkooz G., Rowley C.W. (2012). Turbulence, Coherent Structures, Dynamical Systems And Symmetry.

[9] Berkooz G., Holmes P., Lumley J.L. (1993). The proper orthogonal decomposition in the analysis of turbulent flows. Annu. Rev. Fluid Mech..

[10] Taira K., Brunton S.L., Dawson S.T., Rowley C.W., Colonius T., McKeon B.J., Schmidt O.T., Gordeyev S., Theofilis V., Ukeiley L.S. (2017). Modal analysis of fluid flows: An overview. AIAA J..

[11] Jayaraman B., Lu C., Whitman J., Chowdhary G. (2018). Sparse Convolution-based Markov Models for Nonlinear Fluid Flows. arXiv.

[12] Jayaraman B., Lu C., Whitman J., Chowdhary G. (2019). Sparse feature map-based Markov models for nonlinear fluid flows. Comput. Fluids.

[13] Rowley C.W., Dawson S.T. (2017). Model reduction for flow analysis and control. Annu. Rev. Fluid Mech..

[14] Puligilla S.C., Jayaraman B. (2018). Neural Networks as Globally Optimal Multilayer Convolution Architectures for Learning Fluid Flows. arXiv.

[15] Puligilla S.C., Jayaraman B. Deep multilayer convolution frameworks for data-driven learning of fluid flow dynamics. Proceedings of the 2018 Fluid Dynamics Conference.

[16] Lu C., Jayaraman B. Data-driven modeling for nonlinear fluid flows. Proceedings of the 23rd AIAA Computational Fluid Dynamics Conference.

[17] Brunton S.L., Proctor J.L., Tu J.H., Kutz J.N. (2015). Compressed sensing and dynamic mode decomposition. J. Comput. Dyn..

[18] Bai Z., Wimalajeewa T., Berger Z., Wang G., Glauser M., Varshney P.K. (2014). Low-dimensional approach for reconstruction of airfoil data via compressive sensing. AIAA J..

[19] Bright I., Lin G., Kutz J.N. (2013). Compressive sensing based machine learning strategy for characterizing the flow around a cylinder with limited pressure measurements. Phys. Fluids.

[20] Fukami K., Fukagata K., Taira K. (2019). Super-resolution reconstruction of turbulent flows with machine learning. J. Fluid Mech..

[21] Candès E.J. Compressive sampling. Proceedings of the International Congress of Mathematicians.

[22] Tropp J.A., Gilbert A.C. (2007). Signal recovery from random measurements via orthogonal matching pursuit. IEEE Trans. Inf. Theory.

[23] Candès E.J., Wakin M.B. (2008). An introduction to compressive sampling. IEEE Signal Process Mag..

[24] Needell D., Tropp J.A. (2009). CoSaMP: Iterative signal recovery from incomplete and inaccurate samples. Appl. Comput. Harmon. Anal..

[25] Bui-Thanh T., Damodaran M., Willcox K. (2004). Aerodynamic data reconstruction and inverse design using proper orthogonal decomposition. AIAA J..

[26] Willcox K. (2006). Unsteady flow sensing and estimation via the gappy proper orthogonal decomposition. Comput. Fluids.

[27] Venturi D., Karniadakis G.E. (2004). Gappy data and reconstruction procedures for flow past a cylinder. J. Fluid Mech..

[28] Gunes H., Sirisup S., Karniadakis G.E. (2006). Gappy data: To Krig or not to Krig?. J. Comput. Phys..

[29] Gunes H., Rist U. (2008). On the use of kriging for enhanced data reconstruction in a separated transitional flat-plate boundary layer. Phys. Fluids.

[30] Everson R., Sirovich L. (1995). Karhunen–Loeve procedure for gappy data. JOSA A.

[31] Chaturantabut S., Sorensen D.C. (2010). Nonlinear model reduction via discrete empirical interpolation. SIAM J. Sci. Comput..

[32] Dimitriu G., Ştefănescu R., Navon I.M. (2017). Comparative numerical analysis using reduced-order modeling strategies for nonlinear large-scale systems. J. Comput. Appl. Math..

[33] Zimmermann R., Willcox K. (2016). An accelerated greedy missing point estimation procedure. SIAM J. Sci. Comput..

[34] Saini P., Arndt C.M., Steinberg A.M. (2016). Development and evaluation of gappy-POD as a data reconstruction technique for noisy PIV measurements in gas turbine combustors. Exp. Fluids.

[35] Schmid P.J. (2010). Dynamic mode decomposition of numerical and experimental data. J. Fluid Mech..

[36] Hanagud S., de Noyer M.B., Luo H., Henderson D., Nagaraja K. (2002). Tail buffet alleviation of high-performance twin-tail aircraft using piezostack actuators. AIAA J..

[37] Cohen K., Siegel S., McLaughlin T. Sensor placement based on proper orthogonal decomposition modeling of a cylinder wake. Proceedings of the 33rd AIAA Fluid Dynamics Conference and Exhibit.

[38] Barrault M., Maday Y., Nguyen N.C., Patera A.T. (2004). An ‘empirical interpolation’method: Application to efficient reduced-basis discretization of partial differential equations. C. R. Math..

[39] Yildirim B., Chryssostomidis C., Karniadakis G. (2009). Efficient sensor placement for ocean measurements using low-dimensional concepts. Ocean Model..

[40] Manohar K., Brunton B.W., Kutz J.N., Brunton S.L. (2018). Data-Driven Sparse Sensor Placement for Reconstruction: Demonstrating the Benefits of Exploiting Known Patterns. IEEE Control Syst..

[41] Sarrate R., Nejjari F., Blesa J. (2017). Sensor placement for monitoring. Real-Time Monitoring and Operational Control of Drinking-Water Systems.

[42] Cugueró-Escofet M.À., Puig V., Quevedo J. (2017). Optimal pressure sensor placement and assessment for leak location using a relaxed isolation index: Application to the Barcelona water network. Control Eng. Pract..

[43] Sela L., Amin S. (2018). Robust sensor placement for pipeline monitoring: Mixed integer and greedy optimization. Adv. Eng. Inform..

[44] Huang G.B., Zhu Q.Y., Siew C.K. (2006). Extreme learning machine: Theory and applications. Neurocomputing.

[45] Huang G.B., Wang D.H., Lan Y. (2011). Extreme learning machines: A survey. Int. J. Mach. Learn. Cybern..

[46] Zhou H., Huang G.B., Lin Z., Wang H., Soh Y.C. (2015). Stacked extreme learning machines. IEEE Trans. Cybern..

[47] Al Mamun S., Lu C., Jayaraman B. (2018). Extreme learning machines as encoders for sparse reconstruction. Fluids.

[48] Jayaraman B., Al Mamun S., Lu C. (2019). Interplay of Sensor Quantity, Placement and System Dimension in POD-Based Sparse Reconstruction of Fluid Flows. Fluids.

[49] Tarantola A. (2005). Inverse Problem Theory and Methods for Model Parameter Estimation.

[50] Arridge S.R., Schotland J.C. (2009). Optical tomography: Forward and inverse problems. Inverse Prob..

[51] Tarantola A., Valette B. (1982). Generalized nonlinear inverse problems solved using the least squares criterion. Rev. Geophys..

[52] Neelamani R. (2004). Inverse problems in image processing. Ph.D. Thesis.

[53] Mallet S. (1998). A Wavelet Tour of Signal Processing.

[54] Donoho D.L. (2006). Compressed sensing. IEEE Trans. Inf. Theory.

[55] Baraniuk R.G. (2007). Compressive sensing [lecture notes]. IEEE Signal Process Mag..

[56] Baraniuk R.G., Cevher V., Duarte M.F., Hegde C. (2010). Model-based compressive sensing. IEEE Trans. Inf. Theory.

[57] Sarvotham S., Baron D., Wakin M., Duarte M.F., Baraniuk R.G. Distributed compressed sensing of jointly sparse signals. Proceedings of the Asilomar Conference on Signals, Systems, and Computers.

[58] Candès E.J., Romberg J., Tao T. (2006). Robust uncertainty principles: Exact signal reconstruction from highly incomplete frequency information. IEEE Trans. Inf. Theory.

[59] Candes E.J., Romberg J.K., Tao T. (2006). Stable signal recovery from incomplete and inaccurate measurements. Commun. Pure Appl. Math..

[60] Candes E.J., Tao T. (2006). Near-optimal signal recovery from random projections: Universal encoding strategies?. IEEE Trans. Inf. Theory.

[61] Candes E.J., Romberg J.K. (2005). Signal recovery from random projections. Computational Imaging III.

[62] Chen S.S., Donoho D.L., Saunders M.A. (2001). Atomic decomposition by basis pursuit. SIAM Rev..

[63] Tibshirani R. (1996). Regression shrinkage and selection via the lasso. J. R. Stat. Soc. Ser. B (Methodol.).

[64] Brunton S.L., Proctor J.L., Kutz J.N. (2016). Discovering governing equations from data by sparse identification of nonlinear dynamical systems. Proc. Natl. Acad. Sci. USA.

[65] Sirovich L. (1987). Turbulence and the dynamics of coherent structures. I. Coherent structures. Q. Appl. Math..

[66] Zhou H., Soh Y.C., Jiang C., Wu X. Compressed representation learning for fluid field reconstruction from sparse sensor observations. Proceedings of the 2015 International Joint Conference on Neural Networks (IJCNN).

[67] Kasun L.L.C., Zhou H., Huang G.B., Vong C.M. (2013). Representational learning with extreme learning machine for big data. IEEE Intell. Syst..

[68] Candes E., Romberg J. (2007). Sparsity and incoherence in compressive sampling. Inverse Prob..

[69] Csató L., Opper M. (2002). Sparse on-line Gaussian processes. Neural Comput..

[70] Kubrusly C.S., Malebranche H. (1985). Sensors and controllers location in distributed systems—A survey. Automatica.

[71] Chaturantabut S., Sorensen D.C. (2012). A state space error estimate for POD-DEIM nonlinear model reduction. SIAM J. Numer. Anal..

[72] Trefethen L.N., Bau D. (1997). Numerical Linear Algebra.

[73] Cantwell C.D., Moxey D., Comerford A., Bolis A., Rocco G., Mengaldo G., De Grazia D., Yakovlev S., Lombard J.E., Ekelschot D. (2015). Nektar++: An open-source spectral/hp element framework. Comput. Phys. Commun..

